# Transcriptome and volatile compounds analyses of floral development provide insight into floral scent formation in *Paeonia lactiflora* ‘Wu Hua Long Yu’

**DOI:** 10.3389/fpls.2024.1303156

**Published:** 2024-02-16

**Authors:** Qian Zhao, Min Zhang, Lina Gu, Zihan Yang, Yuqing Li, Jianrang Luo, Yanlong Zhang

**Affiliations:** ^1^ College of Landscape Architecture and Arts, Northwest A&F University, Xianyang, China; ^2^ National Engineering Research Center for Oil Peony, Northwest A&F University, Xianyang, China

**Keywords:** herbaceous peony, floral fragrance, transcriptome, volatiles, monoterpenes

## Abstract

Herbaceous peony (*Paeonia lactiflora*) is a well-known ornamental plant in China, celebrated for its beautiful flowers that can emit fragrances. However, exact molecular mechanisms governing synthesis of floral volatiles within herbaceous peony remain unclear. To address this gap in knowledge, our study focused on analyzing the transcriptome and the levels of floral volatile compounds in *P. lactiflora* ‘Wu Hua Long Yu’ at different stages of flower development. Using gas chromatography-mass spectrometry (GC-MS), we obtained eighteen major volatile compounds, with monoterpenes being the dominant components among them. Our transcriptome analysis, based on pooled sequencing data, revealed the most differentially expressed genes (DEGs) existed between stages S1 and S3 of flower development. Among these DEGs, we identified 89 functional genes associated with the synthesis of volatile monoterpenes, with 28 of these genes showing a positive correlation with the release of monoterpenes. Specifically, key regulators of monoterpene synthesis in herbaceous peony appear to be 1-deoxy-D-xylulose 5-phosphate synthase (DXS), geranyl pyrophosphate synthase (GPPS), and terpene synthase (TPS). Additionally, our study identified some transcription factors (TFs) that may be involved in the biosynthesis of monoterpenes. These discoveries offer invaluable illumination into the intricate molecular underpinnings orchestrating the generation of floral fragrances in herbaceous peonies, and they offer a foundation for further research to identify and utilize candidate gene resources for this purpose.

## 1 Introduction

Floral fragrance, composed of small volatile compounds, serves a vital role in the evolutionary strategies of flowering plants. It facilitates the attraction of pollinators, helping plants combat both biotic and abiotic stresses, thus establishing a unique communication channel between plantae and animalia (Galliot et al., 2006; Knudsen et al., 2006; De Vega et al., 2014; Muhlemann et al., 2014). Furthermore, the aroma is a significant factor in the assessment of ornamental flowers. In recent years, fragrance has gained prominence as a subject of research in the realm of ornamental plants (Fan et al., 2006; Nakamura et al., 2006; Feng et al., 2008; Cao et al., 2009; Zhao et al., 2010; Xu et al., 2012; Zhang et al., 2013; Sun et al., 2015; Kong et al., 2017). To date, about 1,700 floral volatile compounds have been discovered (Knudsen et al., 2006). These compounds are taxonomically categorized into three primary classes contingent upon their biogenic origins: fatty acid derivatives, phenylpropanoids/benzenoids, and terpenoids (Dudareva and Pichersky, 2000).

Terpenoids, the most abundant and diverse class of plant volatiles, are derived from two common isoprene precursors, namely, isopentenyl pyrophosphate (IPP) and dimethylallyl pyrophosphate (DMAPP). Terpenoids are pivotal in determining the rich diversity of floral aromas (Dudareva et al., 2005, 2013; Tholl, 2015). Plants synthesize these isoprene precursors through two distinct pathways: the mevalonic acid (MVA) pathway, located in the cytosol, primarily responsible for sesquiterpene production, and the 2-C-methyl-D-erythritol 4-phosphate (MEP) pathway, situated in the plastids, primarily contributing to monoterpenes (Vranova et al., 2013; Tholl, 2015). The MEP pathway in plants commences with 1-deoxy-D-xylulose 5-phosphate (DXP) production from pyruvate & glyceraldehyde-3-phosphate through DXP synthase (DXS) (Tholl, 2015). IPP and DMAPP are generated through enzymatic reactions chain catalyzed by 2-C-methyl-D-erythritol 4-phosphate cytidylyltransferase (MCT), 2-C-methyl-D-erythritol 2, 4-cyclodiphosphate synthase (MDS), 4-diphosphocytidyl-2-C-methyl-D-erythritol kinase (CMK), 1-deoxy-D-xylulose 5-phosphate reductoisomerase (DXR), hydroxymethylbutenyl diphosphate reductase (HDR), and 4-hydroxy-3-methylbut-2-en-1-yl diphosphate synthase (HDS) (Tholl, 2015). Geranyl diphosphate (GPP) is originated from IPP and DMAPP through the action of GPP synthetase (GPPS). GPP serves as a precursor for monoterpene biosynthesis and is transformed into monoterpenes by terpene synthase (TPS) enzymes (Tholl, 2006). The alternative pathway, the MVA pathway located in the cytosol, employs acetyl CoA as the precursor to generate IPP and DMAPP. IPP and DMAPP are further converted into farnesyl pyrophosphate (FPP), which acts as a precursor for sesquiterpenes. Sesquiterpene synthases catalyze the formation of sesquiterpenes from FPP (Cynthia et al., 1996). Numerous genes in both pathways have been shown to participate in terpenoid biosynthesis. For instance, the *Arabidopsis thaliana* DXS gene was found to significantly enhance monoterpenes’ content in transgenic spike lavender oil (Munoz-Bertomeu et al., 2006). Gao et al. (2018) identified that *FhTPS1* and *FhTPS2* promote monoterpenes synthesis, while *FhTPS8* facilitates sesquiterpenes production. Similarly, in *Osmanthus fragrans*, *OfTPS1, OfTPS2*, and *OfTPS3* were found to promote the synthesis of linalool and trans-β-ocimene, the primary fragrance components (Zeng et al., 2016).

Herbaceous peony, a traditional flower in China, boasts a rich cultivation history spanning over 1500 years (Yang et al., 2020a). Renowned for its elegant petals and a vibrant array of colors, many herbaceous peony cultivars also exhibit the delightful feature of fragrance. In recent years, there has been a heightened emphasis on investigating the composition of floral scents released by specific herbaceous peony cultivars (Feng et al., 2016; Song and Yu, 2017; Myung Suk et al., 2018; Song et al., 2018). Nevertheless, precise mechanisms governing the emission of floral scents in herbaceous peony remain a subject of uncertainty.

RNA sequencing (RNA-seq) serves as the technique for accurately and comprehensively analyzing gene expression transcripts. Iso-Seq, short for Isoform Sequencing, is a third-generation sequencing method that utilizes Single Molecule Real-Time (SMRT) technology. Iso-Seq offers advantages over Illumina RNA-seq by eliminating the need for read length assembly (Eid, 2009; Sharon et al., 2013). In our study, we employed *P. lactiflora* ‘Wu Hua Long Yu’, a fragrant variety, and conducted volatile compounds analysis within its petals utilizing headspace solid-phase microextraction-gas chromatography-mass spectrometry (HS-SPME-GC-MS). We harnessed both Illumina RNA-seq and Pacbio Iso-Seq to establish a database, allowing us to identify candidate genes associated with floral scent production. Our analysis of floral volatile compounds highlighted the prevalence of monoterpenes as the dominant components. Transcriptome analysis unveiled numerous functional genes and transcription factors (TFs) that exhibited a positive correlation with the release of monoterpenes. These findings collectively represent a significant contribution to the comprehension of the mechanisms underpinning fragrance emission in herbaceous peonies, paving the way for further investigations in this field.

## 2 Materials and methods

### 2.1 Plant materials

*P.lactiflora* ‘Wu Hua Long Yu’ plants got cultivated within the fields of Northwest Agriculture and Forestry University, located in Yangling, Shanxi, China. Petal samples were collected at five distinct developmental stages, denoted as S1 (bud stage), S2 (half-opened stage), S3 (full-opened stage), S4 (two days after S3), and S5 (six days after S3, when the petals had fallen). At the S1 stage, the bracts at the top of the bud began to open, and the petals started displaying their coloration. The S2 stage marked the transition from the bud stage to the half-opened stage, commencing on the first day the flowers showed this change. The S3 period commenced on the day the flowers progressed from half-open stage to full-open stage. Interval between S1 and S3 was three days, while the transition from S2 to S3 took just one day. To precisely track the developmental stages of the flowers, each bud was affixed with a labeled card, noting the time and specific stage. To ensure that the flowers were not in a closed state, the samples were collected between 10:00 and 11:00 in the morning. The collected petals were divided into two groups: fresh samples and frozen samples. The fresh sample group was immediately placed in an icebox for subsequent volatile compounds analysis via HS-SPME-GC-MS. Frozen sample group was promptly immersed in liquid nitrogen, preserving it at an ultra-low temperature of -80°C for subsequent transcriptome profiling via RNA-seq.

### 2.2 HS-SPME-GC–MS

For volatile compounds analysis within the petals, we collected 0.5 grams of petals and placed them in a 40 ml transparent glass vial, which was then sealed with a tin-foil septum. The absorption of volatile compounds was achieved using HS-SPME with a fiber coated in 65 μm divinylbenzene/polydimethylsiloxane (DVB/PDMS). Subsequently, fiber got introduced into a GC-MS system (ISQ & TRACE GC Ultra, Thermo Fisher Scientific, USA) for the analysis of volatile components. The injector was maintained at a temperature of 250°C, following the method offered by Luo et al. (2020). The GC conditions were set as follows: a capillary column DB-5MS (30 m × 0.25 mm × 0.25 µm, Agilent) was used. The initial oven temperature was held at 40°C for a period of 2.5 minutes. Subsequently, it was elevated to 200°C at a rate of 5°C·min^-^¹ and retained for an additional 2.5 minutes before further increment to 270°C at a rate of 10°C·min^-^¹, where it was maintained for 5 minutes. Helium of high purity (99.999%) served as the carrier gas, flowing at a rate of 1 ml·min^-^¹. MS analysis was conducted under the following conditions: employing ionization mode EI with an 70 eV electron energy. Ion source temperature was set at 240°C, and the mass scan range encompassed 35-550 amu.

### 2.3 Analysis of volatile compounds

We employed 3-octanal (0.41 mg·ml^-^¹) as an internal standard, and each specimen underwent triplicate analysis. Identification of volatile compounds within each sample got carried out by cross-referencing the National Institute of Standards and Technology (NIST) library and pertinent literature. Quantification of each component was accomplished utilizing the internal standard method. The formula is as follows. Each component content (ng·g^-^¹) = (peak area of each component/peak area of internal standard) × internal standard content (ng·µL^-^¹) ×internal standard volume (µL)/sample weight (g) (Zhao et al., 2023).

### 2.4 RNA extraction, library construction, and sequencing

Total RNA extraction got conducted from the petals of the S1, S2, and S3 developmental stages, with each stage encompassing three biological replicates. Petal tissues were grinded using Trizol reagent (Invitrogen, Carlsbad, CA, USA) on dry ice, the operation standard was referred form professional protocol. Quality and integrity of isolated RNA got evaluated via Agarose gel electrophoresis, RNA Integrity Number (RIN) was ascertained with Agilent 2100 instrument (Agilent Technologies, Palo Alto, CA, USA). Additionally, RNA purity and concentration were detected utilizing Nanodrop micro-spectrophotometer from Thermo Fisher Scientific.

Upon total RNA extracted, mRNA enrichment got achieved employing Oligo(dT) magnetic beads. For Illumina RNA-seq, enriched mRNA underwent fragmentation into shorter sequences utilizing fragmentation buffer. These fragments were subsequently transcribed into cDNA with random primers. Second-strand cDNA synthesis was facilitated by incorporating DNA polymerase I, RNase H, dNTPs, the corresponding buffer. The ensuing cDNA fragments got purified utilizing QiaQuick PCR extraction kit (Qiagen, Venlo, The Netherlands), followed by end repair, poly(A) addition, and ligation to Illumina sequencing adapters. Ligation products were selectively sized through agarose gel electrophoresis, succeeded by PCR amplification. Ultimately, sequencing was executed on the Illumina HiSeqTM 4000 platform (Lei et al., 2018).

For Pacbio ISO-seq, the process commenced with the enrichment of mRNA, which was subsequently subjected to reverse transcription using the Clontech SMARTer PCR cDNA Synthesis Kit. The meticulous PCR cycle count was applied in the generation of double-stranded cDNA. Subsequently, a large-scale PCR was executed to facilitate the subsequent construction of SMRTbell libraries. The cDNA molecules underwent a sequence of essential procedures, encompassing DNA damage repair, end repair, and ligation to sequencing adapters. The resulting SMRTbell templates were annealed to sequencing primers, polymerase-bound, and sequenced utilizing the PacBio Sequel II platform (Wan et al., 2019).

### 2.5 Transcriptome analysis

For Illumina RNA-seq data analysis, we initiated the data preprocessing by employing the fastp version 0.18.0 tool, as expounded by Chen et al. (2018). The specific parameters employed in this process included the excision of reads containing adapters or those with more than 10% undetermined nucleotides, and the elimination of low-quality reads containing over 50% of bases with a Q-value of 20 or lower.

When handling Pacbio ISO-seq data, a dedicated analytical pipeline tailored for isoform sequencing was employed, underpinned by Pacific Biosciences technology. The workflow commenced with the extraction of high-fidelity circular consensus sequences (CCS) from subreads BAM files. Transcript integrity was rigorously assessed by confirming the presence of 5’ and 3’ primers, as well as the polyA tail within CCS reads. Only reads exhibiting these structural characteristics were designated as full-length (FL) reads. Subsequently, the production of full-length non-chimeric (FLNC) reads was achieved through the removal of primers and barcodes, the trimming of polyA tails, and the concatenation of full passes. Comprehensive isoform sequences were constructed by clustering FLNC reads. Similar FLNC reads underwent a hierarchical clustering process using minimap2 to generate unpolished consensus isoforms. These unpolished consensus isoforms were further enhanced using the Quiver algorithm. Isoforms with a prediction accuracy exceeding or equal to 0.99 were selected for downstream sequence analysis. Concurrently, based on the Illumina RNA-seq data, these high-quality isoforms were subjected to correction using the LoRDEC software. The ultimate collection of high-quality, full-length, polished consensus sequences was obtained by eliminating redundancy with the CD-HIT software.

The culminating isoforms underwent annotation through the utilization of the BLASTx program against five reference databases, with an E-value threshold set at 1e-5. These databases encompassed the NCBI non-redundant protein (Nr), Swiss-Prot protein, KEGG, GO, and Cluster of Orthologous Groups of proteins (COG/KOG). To estimate gene expression levels, the high-quality and clean reads from Illumina RNA-seq were aligned to the reference transcriptome derived from Pacbio ISO-seq using RSEM (Li and Dewey, 2011). Gene abundances were subsequently quantified and normalized to yield RPKM values (Li and Dewey, 2011).

### 2.6 Identification and analysis of DEGs

In our study, we conducted differential expression analysis by leveraging the DESeq2 software, adhering to the established protocols delineated by Love et al. (2014) and Robinson et al. (2010). Genes that met the stringent criteria of |log2 fold change (FC)| > 1 and a FDR threshold of < 0.05 were subsequently designated as DEGs. Those DEGs exhibiting congruent expression patterns were subjected to hierarchical grouping. To elucidate the functional implications of these DEGs, we carried out GO and KEGG pathway enrichment analyses. For the GO enrichment analysis, we harnessed the GOseq software, while the KEGG pathway enrichment analysis was executed via the KOBAS platform (Mao et al., 2005; Young et al., 2010).

Further, we employed the Short Time-series Expression Miner (STEM) software for the purpose of clustering the DEGs based on their dynamic expression patterns (Xiong et al., 2021). The software received the expression levels of all DEGs as input, and specific parameters were set (-pro 20 -ratio 1.0000, where log2(2) = 1, log2(1.5) = 0.5849625, and log2(1.2) = 0.2630344). Subsequently, we performed hypothesis testing to calculate p-values. For the prediction of TF families, we utilized Hmmscan to align the protein-coding sequences of isoforms against the Plant TFdb (http://planttfdb.cbi.pku.edu.cn/).

### 2.7 Quantitative real-time PCR validation

The qRT-PCR analysis was conducted using TB Green^®^ Premix Ex Taq™ II (RR820A, Takara, Dalian, China) on a Step One Plus Real-Time PCR system (Applied Biosystems, USA). Each 20 μL reaction volume encompassed a composition of 10 μL TB Green Premix Ex Taq II, 0.8 μL of cDNA, 0.8 μL of each primer (10 μM), 0.4 μL of ROX, and 7.2 μL of double-distilled water (ddH2O). The qRT-PCR program adhered to the following protocol: an initial denaturation phase at 95°C for 30 seconds, executed once, was followed by 40 cycles, each comprising denaturation at 95°C for 3 seconds and annealing/extension at 60°C for 30 seconds. Subsequently, a final melt curve analysis was performed, involving denaturation at 95°C for 15 seconds, annealing at 60°C for 1 minute, and a final denaturation at 95°C for 15 seconds. To determine the relative expression levels of the genes, the 2^-△△CT^ method, as described by Livak and Schmittgen (2001), was applied, with GAPDH (Li, 2017) employed as the reference gene. Detailed information regarding the specific primer sequences for the chosen isoforms can be located in **Supplementary Table S1**.

### 2.8 Statistical analysis

All experiments got completed with three biological replicates to ensure the robustness of the results. Statistical significance was determined via Duncan’s multiple range test, with a predefined threshold of significance set at p < 0.05. Venn diagrams and expression heatmaps were crafted by the omicshare platform (https://www.omicshare.com). Pearson correlations between variables were calculated using the SPSS version 26.0 software. Protein-Protein Interaction (PPI) analysis was carried out utilizing data from STING database. The correlation and PPI networks were visualized and plotted using the Cytoscape software (v3.8.2).

## 3 Results

### 3.1 Variations of volatile compounds during floral development

**Figure 1:**
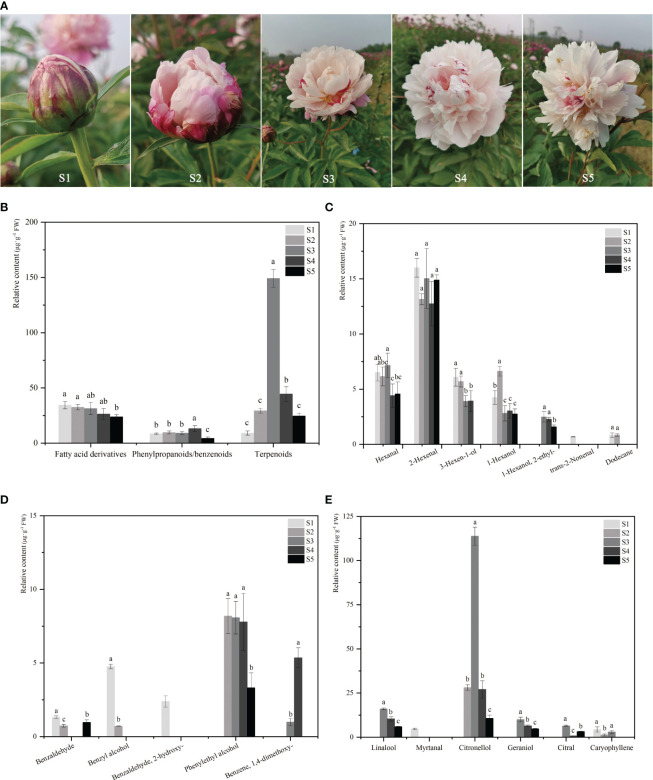
The phenotype and floral volatile compound levels of *P. lactiflora* ‘Wu Hua Long Yu’ during five development stages. **(A)** The phenotype of *P. lactiflora* ‘Wu Hua Long Yu’ at five stages. **(B)** The content of three classes of volatile compounds released from five stages. **(C)** The content of 7 fatty acid derivatives at five stages. **(D)** The content of 5 phenylpropanoids/benzenoids at five stages. **(E)** The content of 6 terpenoids at five stages. Data represent means and standard errors of three replicates. The different letters above the columns indicate significant (p < 0.05) differences according to Duncan’s multiple range test. The detailed data of volatile compounds were shown in the **Supplementary Table S2**.

The developmental stages of *P. lactiflora* ‘Wu Hua Long Yu’ are depicted in **Figure 1A**. From a sensory perspective, no fragrance is detected during the S1 stage, while a subtle fragrance emerges in the S2 stage, reaching its peak intensity during the S3 stage. As the flowers fully open (S4), the fragrance begins to diminish, and by the time the petals wither (S5), the fragrance becomes even fainter. To investigate the emission profile of floral scent during the development of herbaceous peony flowers, volatile compounds emitted from the petals of ‘Wu Hua Long Yu’ at five different stages were sampled using HS-SPME and analyzed by GC-MS. A total of 18 major volatile compounds, comprising six terpenes, five phenylpropanoids/benzenoids, and seven fatty acid derivatives, were identified in these five stages, with 11, 10, 12, 11, and 10 major volatiles detected in S1, S2, S3, S4, and S5, respectively (**Supplementary Table S2**).

The analysis of the total volatile compound release demonstrated a pattern that closely mirrored sensory perception, with an initial increase followed by a decline (**Figure 1B**). The total release of volatile components continuously increased from S1 to S3 before beginning to decrease. This trend aligns with previous studies on the release of volatile compounds during flower opening (Xu et al., 2012; Zhang et al., 2021). Specifically, the release pattern of terpenes mirrored that of the total volatile components. Terpenes exhibited minimal release during S1, followed by a steady increase, with the highest release occurring at S3 (149.02 μg·g^-^¹). The content of fatty acid derivatives displayed a decreasing trend and remained relatively stable among the S1 to S4 stages. Phenylpropanoids/benzenoids showed no significant differences in release among the first three stages, with the highest content observed in S4 and a gradual decrease in S5 (**Figure 1B**).

Among the three types of volatile compounds, only terpenoids exhibited a release pattern consistent with sensory perception. Terpenoids had the highest total release amount and proportion, and among them, only caryophyllene was a sesquiterpene with low content. Monoterpenoids were identified as the primary floral aroma compounds in herbaceous peony, contributing to the strong aroma observed during the full-opened stage.

The release patterns of the 18 volatiles were analyzed across the five stages. Fatty acid derivatives and phenylpropanoids/benzenoids were found to have inconsistent release patterns with the perception of floral fragrance (**Figures 1C, D**). In contrast, linalool, citronellol, and geraniol exhibited release patterns that correlated with the sensory perception of fragrance, peaking in S3 and subsequently declining, establishing them as the principal aromatic components of ‘Wu Hua Long Yu’ (**Figure 1E**). Significantly, linalool, citronellol, and geraniol were identified as monoterpenoids, confirming their role as the primary contributors to the fragrance of herbaceous peony (Zhao et al., 2023).

### 3.2 RNA sequencing, gene annotation, and functional classification

To gain insights into release mechanism of volatiles in ‘Wu Hua Long Yu’, we selected three distinct stages (S1, S2, and S3) with significant differences in volatile contents for RNA-seq analysis. Petals from these three stages were used to create nine Illumina RNA-seq libraries, aiming to explore the molecular basis of changes in floral scent in ‘Wu Hua Long Yu’. After the removal of low-quality reads, a total of 65.9 Gb of clean data with Q30 (base quality value ≥ 30) exceeding 92.21% was generated using Illumina HiSeq 4000 (**Supplementary Table S3**). Pearson correlation analysis and principal component analysis (PCA) were skillfully executed to gauge the fidelity of Illumina RNA-seq data (**Supplementary Figure S1**), confirming the robustness of the biological replicates, which supported the suitability of the data for subsequent analyses.

For Pacbio ISO-seq, a total of 21,796,952 subreads (50.7 Gb) were obtained, resulting in 665,658 CCS reads with an average length of 2630 bp following data processing and screening. After clustering and initial correction, a total of 48,796 consensus isoforms were derived. The Pacbio ISO-seq data were corrected based on the Illumina RNA-seq data using LoRDEC. Redundancies were eliminated using CD-HIT software, yielding 37,878 isoforms with an average length of 2425.45 bp. Notably, the N50 value of these isoforms, measuring at 2637 bp, exceeded the calculated average length, serving as a testament to the assembly’s high degree of completeness (**Supplementary Figure S2**; **Supplementary Tables S4**, **S5**).

**Figure 2:**
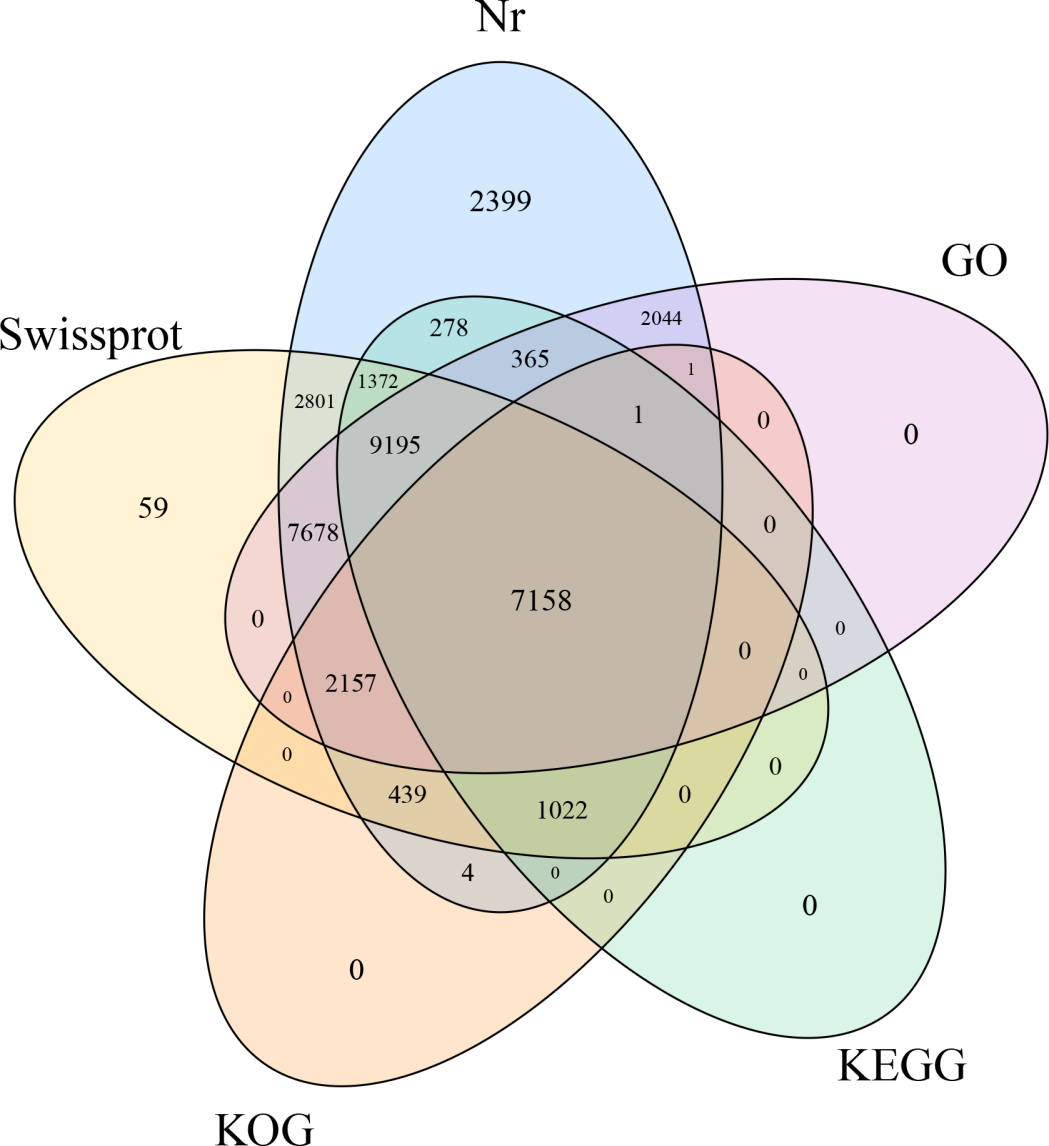
Venn plot of isoform numbers annotated to five databases.

Among the 37,878 isoforms, 36,973 isoforms (97.61%) were annotated through BLASTx searches against five public databases (blast E-value ≤1e-5). Specifically, 36,914 (99.84%), 19,391 (52.45%), 10,782 (29.16%), 31,881 (86.23%), and 28,599 (77.35%) got annotated to Swiss-Prot, KEGG, Nr, GO, and KOG databases, respectively. It’s worth noting that all isoforms from KEGG, KOG, and GO databases, except for 59 isoforms in the Swiss-Prot database, were annotated in the NR database. In total, 7,158 isoforms were annotated across all five databases (**Figure 2**).

The top matches in terms of gene similarity were found with *Vitis vinifera* (8,078, 21.88%), followed by *Actinidia chinensis* (2,228, 6.03%), *Camellia sinensis* (1,884, 5.10%), *Juglans regia* (1,267, 3.43%), and *Quercus lobata* (1,231, 3.33%) (**Supplementary Figure S3**), indicating the highest homology between *P. lactiflora* ‘Wu Hua Long Yu’ and *V. vinifera*.

To functionally characterize the transcriptome isoforms of ‘Wu Hua Long Yu’, a GO analysis was conducted. A total of 28,599 isoforms got categorized into 53 functional groups belonging to GO’s primary categories (24 biological processes, 17 molecular functions, and 12 cellular components) (**Supplementary Figure S4**). The top three of the GO terms among the 24 biological processes: “cellular process”, “metabolic process”, and “single-organism process”.

In the KOG database, 10,782 isoforms were distributed across 25 KOG categories (**Supplementary Figure S5**). “Signal transduction mechanisms” was the most abundant KOG classification, encompassing 2,245 isoforms.

Out of the 19,391 isoforms, 137 pathways from the KEGG database were identified. The top three pathways were “metabolic pathways” (5,099), “biosynthesis of secondary metabolites” (2,965), and “carbon metabolism” (888). Notably, many isoforms were annotated into secondary-metabolism-related pathways, including “phenylpropanoid biosynthesis” (211), “fatty acid biosynthesis” (182), “terpenoid backbone biosynthesis” (156), and “sesquiterpenoid and triterpenoid biosynthesis” (46). Most significantly, 27 isoforms were annotated within “monoterpene biosynthesis” pathway, which is associated with floral fragrance release in ‘Wu Hua Long Yu’ (**Supplementary Table S6**).

### 3.3 DEGs enrichment and expression analyses

To assess the transcriptome completeness and reliability, isoforms obtained from Pacbio ISO-seq were used as a reference. Over 98.27% of the 37,878 isoforms were sequenced and identified in the nine databases (**Supplementary Table S7**). The mapping of Illumina RNA-seq reads to these isoforms resulted in over 86.03% of effective reads being mapped (**Supplementary Table S8**), confirming the pooled RNA-seq data reliability. **Supplementary Figure S6** visually represents the isoform abundance expression in each sample, showing data density at different positions in various samples. This combination of Illumina RNA-seq and Pacbio ISO-seq enabled the creation of a longer and more accurate transcriptome database.

**Figure 3:**
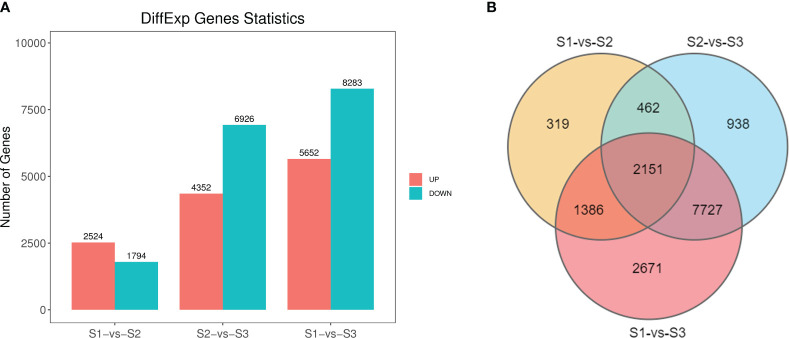
Gene expression comparisons of three stages in *P. lactiflora* ‘Wu Hua Long Yu’. **(A)** Histogram of up- and downregulated genes with the latter as the reference in three comparisons. **(B)** Venn plot of DEGs from three comparisons.

In evaluating the significance of isoforms among different libraries (S1-S3), criteria such as FDR < 0.05 and |log2FC| > 1 were employed. We identified a total of 14,369 DEGs, with 4,318 significant DEGs in the S1 vs S2 comparison group (comprising 2,524 up-regulated and 1,794 down-regulated isoforms), 11,278 DEGs (4,352 up-regulated and 6,926 down-regulated isoforms) in the S2 vs S3 comparison, and 13,935 DEGs (5,652 elevated and 8,283 suppressed isoforms) in the S1 vs S3 comparison (**Figure 3A**). Notably, 319 unique isoforms were identified in S1 vs S2, 938 unique isoforms in S2 vs S3, and 2,671 unique isoforms in S1 vs S3. Moreover, 2,151 isoforms notably differed in expression levels across all three comparison groups (**Figure 3B**).

‘Wu Hua Long Yu’ exhibited considerable differences in fragrance release during the three stages, and these differences were closely associated with the related regulatory DEGs. In summary, the differences observed in the S1 vs S2 and S2 vs S3 comparisons were not as pronounced as in the S1 vs S3 comparison. ‘Wu Hua Long Yu’ began to release fragrance during S2, and fragrance release increased significantly after the flowers fully opened, aligning with the release patterns of volatile compounds during these three stages.

The DEGs identified in the S1 vs S2, S2 vs S3, and S1 vs S3 comparisons were annotated into reference terms based on the GO and KEGG databases to elucidate the differences in biological processes during petal development. For the GO database, a total of 22,400 DEGs were annotated (**Supplementary Figure S7**). Notably, DEGs related to the “terpenoid biosynthetic process” (GO: 0016114) numbered 73, 101, and 174 in the S1 vs S2, S2 vs S3, and S1 vs S3 comparisons, respectively. This finding aligns with the release trend of terpenoids from S1 to S3.

For the KEGG database, 1,319 DEGs were mapped to 122 KEGG pathways in the S1 vs S2 comparison, 3,427 DEGs were associated with 133 KEGG pathways in the S2 vs S3 comparison, and 4,260 DEGs were annotated to 134 KEGG pathways in the S1 vs S3 comparison (**Supplementary Figure S8**). Among these pathways, several biosynthesis pathways relevant to floral fragrance were identified, including “sesquiterpenoid and triterpenoid biosynthesis” (ko00909), “terpenoid backbone biosynthesis” (ko00900), “Fatty acid biosynthesis” (ko00061), “phenylpropanoid biosynthesis” (ko00940), and “monoterpenoid biosynthesis” (ko00902). In the S1 vs S2 comparison group, DEGs involved in ko00061, ko00900, ko00902, ko00909, and ko00940 numbered 25, 32, 9, 26, and 59, respectively. In the S2 vs S3 comparison, DEGs in these five pathways were 65, 69, 27, 38, and 145, respectively, while the S1 vs S3 comparison group had 119, 101, 27, 39, and 163 DEGs, respectively. The DEGs associated with ko00061, ko00900, ko00902, ko00909, and ko00940 in S1 vs S3 were generally superior than those in the other two groups, consistent with the changes in floral volatiles among the three stages. As monoterpenoids were the primary components of ‘Wu Hua Long Yu’s’ fragrance, there were more DEGs in the ko00900 and ko00902 pathways in S2 vs S3 and S1 vs S3 than in S1 vs S2. The ko00909 pathway exhibited no significant changes in DEGs, supporting the notion that a substantial number of monoterpenoids were synthesized in the S3 period. These enrichments provide valuable resources for studying dynamic changes in specific biological processes involved in the biosynthesis of the floral scent of herbaceous peony.

### 3.4 Expression pattern analysis of DEGs

To identify specific gene sets exhibiting distinct expression patterns, such as continuous increases in expression, an analysis of data trends across three different periods was conducted. Clustering results were obtained to categorize genes based on their expression patterns. Subsequently, the gene data were normalized according to these trends, and individual trend pattern figures were generated.

**Figure 4:**
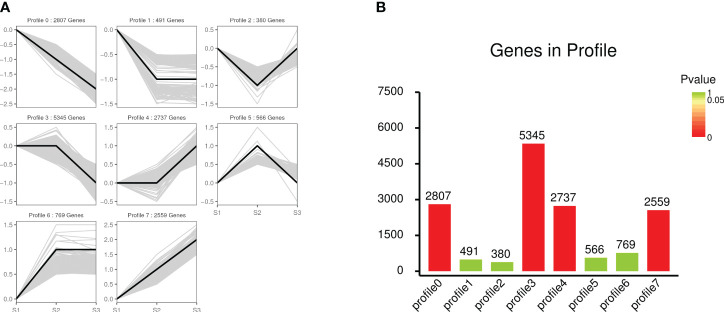
STEM analysis of gene expression profile. **(A)** The trend of all gene expression patterns. The black line represented the expression tendency of all the genes in each frame, the gray line represents each gene. **(B)** Trend gene number and p-value histogram. The height of the column in the figure represents the number of genes, and the color of the column represents the p-value. The p-value is calculated through hypothesis testing.

A total of 15,654 DEGs at different stages (S1-S3) were classified into eight profiles (profile 0 to profile 7) based on their expression patterns using the STEM software. These profiles consisted of 2,807, 491, 380, 5,345, 2,737, 566, 769, and 2,559 genes, respectively (**Figure 4A**). Go and KEGG analyses got conducted on these eight profiles, as depicted in **Supplementary Figures S9**, **S10**. Among these eight profiles, four exhibited significant expression patterns (profiles 0, 3, 4, and 7) (**Figure 4B**).

Profile 7, one of the significant expression profiles, was selected for further analysis due to its consistent trend with volatile compounds release. In terms of biological processes within the GO database, 55 DEGs were enriched in the “terpenoid biosynthetic process” (GO: 0016114) term. For the KEGG database, a total of 817 DEGs were annotated and categorized into 107 pathways. Notably, the “terpenoid backbone biosynthesis” pathway (ko00900), which contained 45 DEGs, is linked to the synthesis of the fragrance of ‘Wu Hua Long Yu’.

### 3.5 Analysis of DEGs related to terpene biosynthesis

**Figure 5:**
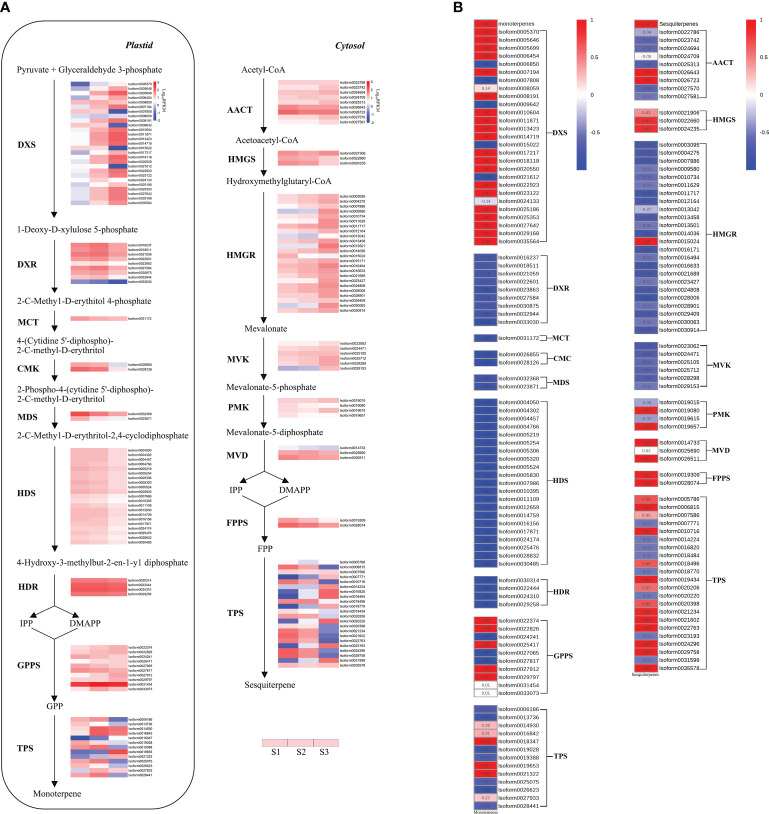
Expression and correlation heatmaps of genes related to terpenoid synthesis of *P. lactiflora* ‘Wu Hua Long Yu’. **(A)** Expression heatmap. Gene expression levels in three stages were represented by color gradations. **(B)** Correlation heatmap between terpene release and gene expression. Correlation levels were represented by color gradations. The detailed data were shown in the **Supplementary Tables S9**, **S10**.

Terpene biosynthesis in plants is typically carried out through two distinct pathways: the MEP pathway in the plastid and the MVA pathway in the cytoplasm. Monoterpenes, for instance, are typically synthesized through seven enzymatic steps within the MEP pathway (Zhang et al., 2021). A total of 66 DEGs related to the MEP pathway were identified (**Figure 5A**). Notably, among these DEGs, twenty DXS genes exhibited similar expression patterns to the release of monoterpenes, displaying strong positive correlations (r > 0.99) with monoterpene content (**Figure 5B**). Additionally, most DXR genes showed an increase from S1 to S2, followed by a decrease in S3. However, other genes involved in the MEP pathway, such as MCT genes, CMK genes, MDS genes, HDS genes, and HDR genes, exhibited a decrease from S1 to S3, displaying an opposing trend to monoterpene release (**Figure 5B**).

In addition to the MEP pathway’s contribution to monoterpenes, sesquiterpenes are typically produced through six enzymatic steps in the MVA pathway (Zhang et al., 2021). A total of 49 DEGs related to the MVA pathway were identified (**Figure 5A**). Among these, two AACT genes, two HMGS genes, one HMGR gene, two PMK genes, and two MVD genes displayed positive correlations (r > 0.83) with sesquiterpene content (**Figure 5B**).

As precursors for monoterpenes and sesquiterpenes, GPP and FPP are produced by GPPS and FPP synthase (FPPS), respectively (Tholl, 2015; Zhang et al., 2021). Ten GPPS and two FPPS genes were found (**Figure 5A**). Among these, five GPPS genes were positively correlated (r > 0.92) with monoterpene content from S1 to S3, while the two FPPS genes were positively correlated (r > 0.91) with sesquiterpene content (**Figure 5B**).

Following the formation of precursors GPP and FPP, terpenoids are synthesized by TPS enzymes that catalyze the respective precursors. GPP is involved in monoterpene synthesis, while FPP is involved in sesquiterpene synthesis through different TPS genes (Tholl, 2006). In total, 13 and 22 TPS genes related to monoterpenes and sesquiterpenes synthesis, respectively, were identified (**Figure 5A**). Among these, three of the 13 mono-TPS genes were positively correlated (r > 0.97) with the content of monoterpenes, while nine of the 22 sesqui-TPS genes exhibited a positive correlation (r > 0.89) with the content of sesquiterpenes (**Figure 5B**).

**Figure 6:**
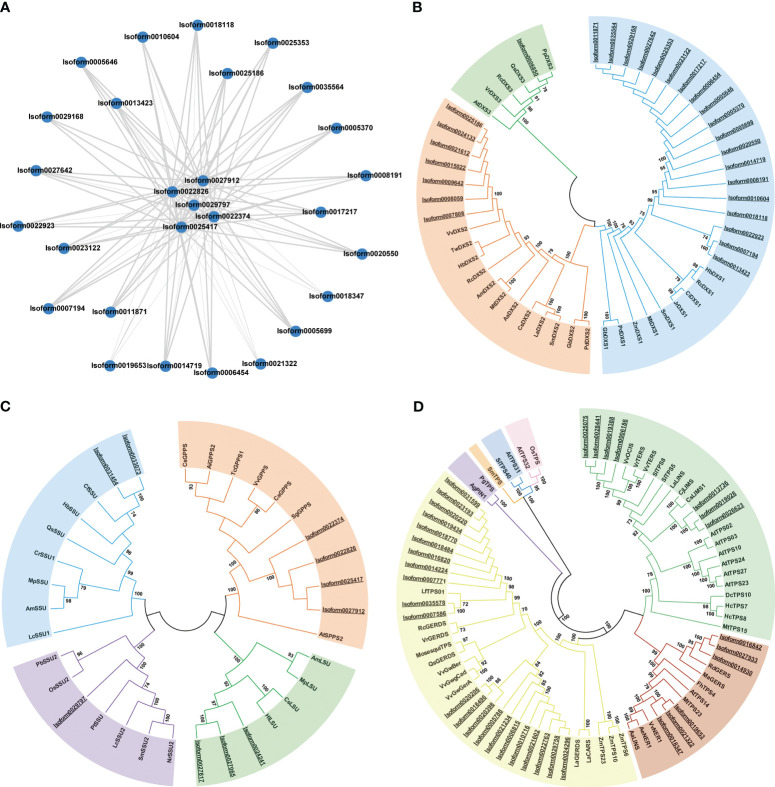
PPI network and phylogenetic analyses of DEGs from *P. lactiflora* ‘Wu Hua Long Yu’. **(A)** The PPI network for the corresponding proteins of 28 DEGs, in which the thickness of the connecting lines is directly proportional to the strength of protein-protein interactions. **(B)** Phylogenetic analysis of DXS proteins. Blue: DXSI clade, orange: DXS II clade, green: DXS III clade. **(C)** Phylogenetic analysis of GPPS proteins. Orange: GPPS clade, blue: GPPS-SSU1 clade, purple: GPPS-SSU2 clade, green: GPPS-LSU clade. **(D)** Phylogenetic analysis of TPS proteins. Yellow: TPS-a, green: TPS-b, blue: TPS-c, purple: TPS-d, pink: TPS-e/f, red: TPS-g, orange: TPS-h. The proteins from *P. lactiflora* ‘Wu Hua Long Yu’ were underlined. The accession numbers used in this analysis were listed in **Supplementary Tables S11**–**S13**.

In summary, more DEGs were associated with monoterpene synthesis compared to the MVA pathway. Some key enzymes involved in both pathways displayed trends similar to the corresponding volatile compounds. Monoterpenoids were identified as a significant aroma component in ‘Wu Hua Long Yu’. A total of 28 DEGs were strongly positively correlated with monoterpene synthesis (including 20 DXSs, five GPPSs, and three TPSs), making them the most significant DEGs. To understand the interactions between these DEGs, a Protein-Protein Interaction (PPI) network was constructed for the corresponding proteins of these 28 DEGs (**Figure 6A**). Notably, among this PPI network, five GPPSs (*Isoform22374*, *Isoform22826*, *Isoform25417*, *Isoform27912*, *Isoform29797*) were identified as the highly connected genes, displaying strong interactions with DXS and TPS proteins, suggesting they play a central role in the biosynthesis of monoterpenoids.

### 3.6 Phylogenetic analysis of DEGs related to monoterpene biosynthesis

Among the genes related to monoterpene synthesis, twenty DXS, five GPPS, and three TPS genes exhibited strong positive correlations (r > 0.92) with the release trend of monoterpenes. All differentially expressed isoforms of DXS, GPPS, and TPS were selected for constructing the phylogenetic tree.

DXS genes were categorized into three subfamilies: DXS I, which is responsible for catalyzing the formation of terpenoid precursor substances; DXS II, which encodes enzymes involved in specific secondary metabolites; and DXS III, which encodes enzymes with unknown or inactivated functions (Walter et al., 2002; Kim et al., 2005). Phylogenetic analysis revealed that 19 DXSs belonged to the DXS I subfamily, and all 19 of these genes were strongly positively correlated with monoterpenoid synthesis. Seven DXSs fell within the DXS II clade, with only *Isoform0025186* showing a strong positive correlation with monoterpenoid synthesis. One DXS belonged to DXS III (**Figure 6B**). These findings suggest that 19 DXSs in the DXS I subfamily and 1 DXS in the DXS II subfamily are likely involved in the aroma synthesis of ‘Wu Hua Long Yu’.

GPPS genes were classified into two types: heterodimeric GPPS and homodimeric GPPS. Heterodimeric GPPS comprises two subunits, the small regulatory subunit (SSU1, SSU2) and the catalytic large subunit (LSU). Both clades were closely related to the release of monoterpenes (Tholl et al., 2004). In our study, four GPPSs belonged to the homodimeric GPPS clade, while six GPPSs belonged to the heterodimeric GPPS subfamily (comprising two GPPS-SSU1, one GPPS-SSU2, and two GPPS-LSU) (**Figure 6C**). The five GPPSs that were positively correlated with the release trend of monoterpenes were classified into homodimeric GPPS and GPPS-SSU2, suggesting that homodimeric GPPS and GPPS-SSU2 play a major role in monoterpenoid synthesis in ‘Wu Hua Long Yu’.

TPS genes were divided into seven subfamilies. Among the TPS genes in ‘Wu Hua Long Yu’, 22 belonged to TPS-a, seven to TPS-b, and six to TPS-g. Notably, the three mono-TPS genes that exhibited positive correlations with the release of monoterpenoids all belonged to the TPS-g subfamily and encoded monoterpene synthetases without the R(R)X8W domain (**Figure 6D**). This suggests that they may promote monoterpene synthesis in the S3 stage (Tholl and Lee, 2011; Gao et al., 2018).

### 3.7 Analysis of differentially expressed TFs

**Figure 7:**
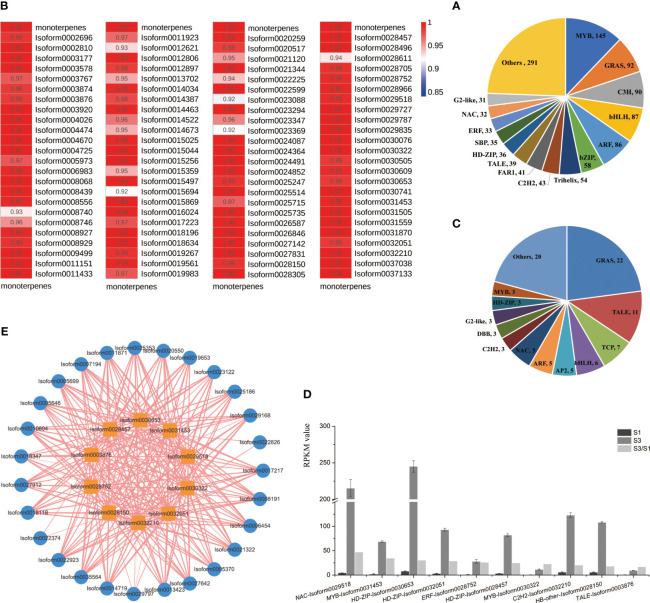
Analyses of differentially expressed TFs. **(A)** Correlation heatmap between monoterpene release and TFs expression in the profile 7. Correlation levels were represented by color gradations. **(B)** Classification of TFs. **(C)** Classification of TFs assigned in the profile 7. **(D)** TFs significantly up-regulated in S3 period from profile 7. **(E)** Correlation network between TFs significantly up-regulated in S3 and 28 DEGs related to terpenoid synthesis. Outer circle: DEGs, inner square: TFs, width of lines: correlation coefficient (0.95-1).

TFs functions crucially within various plant development and physiological processes (Yang et al., 2012). In the ‘Wu Hua Long Yu’ transcriptome, a total of 1,193 differentially expressed TFs were identified and grouped into 80 TF families. The most abundant TF family was MYB (145), followed by GRAS (92), C3H (90), bHLH (87), ARF (86), and bZIP (58) (**Figure 7A**).

To explore the TFs associated with the release of monoterpenes, we focused on the correlation between all transcription factors in profile 7 and the release of monoterpenes, revealing correlations of more than 0.92 (**Figure 7B**). Subsequently, we conducted a detailed analysis of the TFs in profile 7, identifying 96 TFs distributed across 26 TF families (**Figure 7C**).

Among these 96 TFs, when comparing S3 to S1, the top ten significantly up-regulated TFs in the S3 period were identified (**Figure 7D**). These TFs included one NAC (*Isoform0029518*), two MYB (*Isoform0031453*, *Isoform0030322*), three HD-ZIPs (*Isoform0030653*, *Isoform0032051*, *Isoform0028457*), one ERF (*Isoform0028752*), one C2H2 (*Isoform0032210*), one HB-other (*Isoform0028150*), and one TALE (*Isoform0003876*). To further understand their roles, we analyzed the correlations between these ten TFs and the 28 important DEGs mentioned earlier and found that these ten TFs exhibited strong correlations with these DEGs (**Figure 7E**).

**Figure 8:**
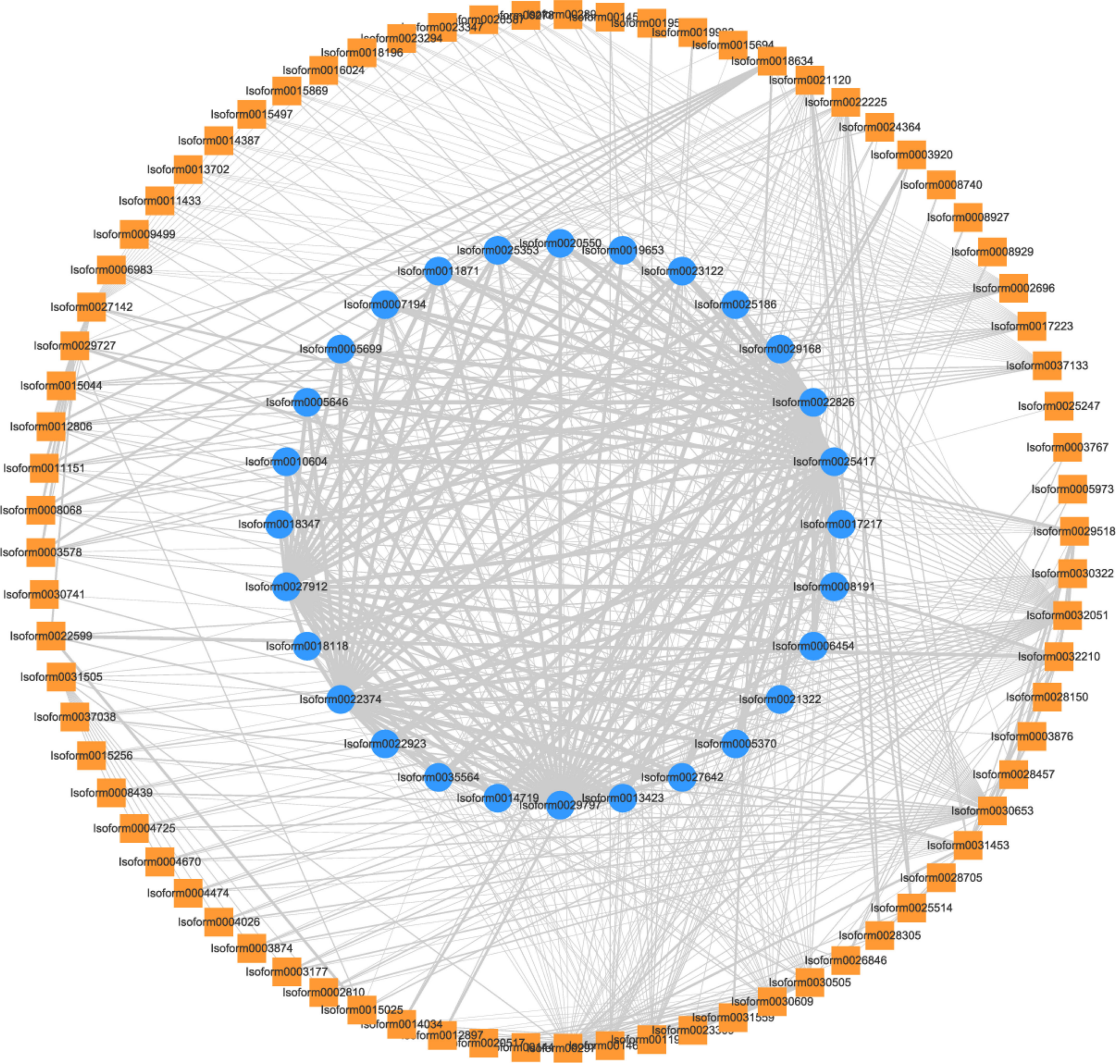
PPI network between TFs in profile 7 and 28 DEGs related to monoterpenoid synthesis. Outer square: TFs, inner circle: DEGs, width of lines: the strength of protein-protein interactions.

Additionally, we constructed a PPI network involving 28 important DEGs and 96 TFs in profile 7. This analysis revealed TFs interacting with DXS and GPPS proteins. Specifically, we identified one NY-FB (*Isoform0030305*), one BBX (*Isoform0030609*), two bHLHs (*Isoform00*11923, *Isoform00*14673) and one CO-like (*Isoform00*29787) with the interactions with DXS proteins. Furthermore, two C3Hs (*Isoform0021120*, *Isoform0022225*) interacted with both DXS and GPPS proteins. These findings suggest that these two C3Hs may play a crucial role in monoterpenoid synthesis, as they can interact with key enzymes involved in terpene biosynthesis, such as DXS and GPPS (**Figure 8**).

### 3.8 The result of qRT-PCR experimental verification

**Figure 9:**
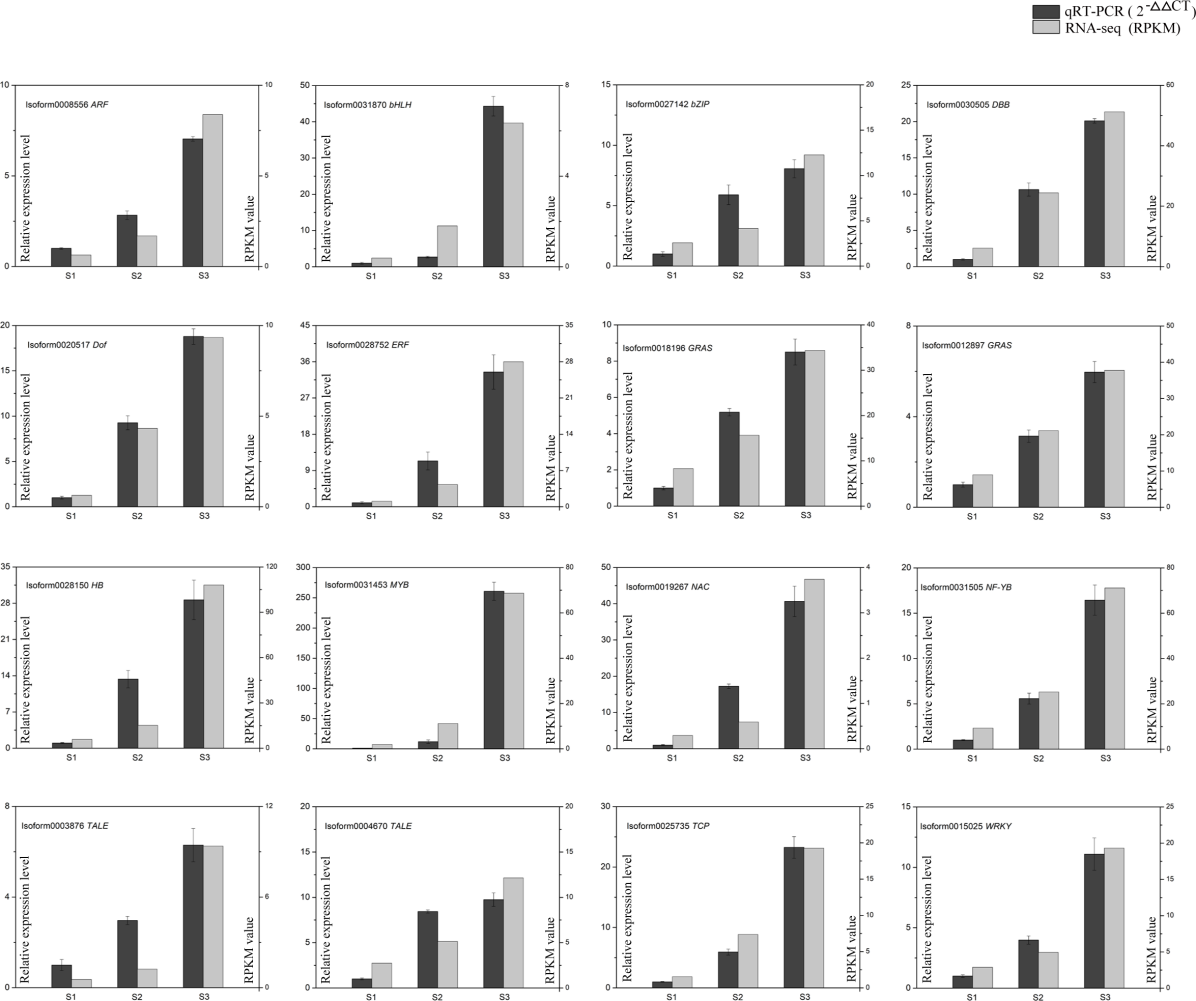
The expression levels of 16 DEGs between qRT-PCR and RNA-seq. Black columns indicate relative expression levels of qRT-PCR (left y-axis). Grey columns indicate expression levels of RNA-Seq (right y-axis). Data represent means and standard errors of three replicates.

To verify the reliability of the RNA-Seq data, a randomly selected set of 16 DEGs was subjected to qRT-PCR analysis. The relative expression levels determined by qRT-PCR were largely consistent with the expression patterns observed in the RNA-Seq data, providing additional support for the credibility and accuracy of the RNA-Seq results (**Figure 9**).

## 4 Discussion

Floral fragrance, primarily composed of low molecular volatile compounds, represents a secondary metabolite released by flowers. The synthesis of aroma components in flowers mainly involves three metabolic pathways: the terpenoids metabolic pathway, benzenoids/phenylpropanoids metabolic pathway, and fatty acid derivatives metabolic pathway (Zhang et al., 2021). Through the analysis of volatile compounds in the petals of *P. lactiflora* ‘Wu Hua Long Yu’ at five floral developmental stages using GC-MS, it was revealed that monoterpenes serve as the predominant volatile compounds in herbaceous peony flowers. Monoterpenes have been identified as the major fragrance components in various plant species, including *Freesia*, *Lilium*, *Magnolia*, and *Genus Hosta* (Zhao et al., 2012; Liu et al., 2015; Yang et al., 2016; Dhandapani et al., 2017; Hu et al., 2013, 2017; Gao et al., 2018). Specifically, monoterpenes such as citronellol, geraniol, and linalool were found to be released in substantial quantities during the S3 developmental stage, serving as the primary contributors to the fragrance of ‘Wu Hua Long Yu’. The advent of RNA-Seq technology has made it feasible to conduct transcriptome studies on plant species lacking a reference genome (Luo et al., 2017). By leveraging Pacbio ISO-Seq technology, which offers extended read lengths conducive for splicing, coupled with thorough error correction and validation through Illumina RNA-Seq data, a high-quality transcriptome dataset was established (Zhao et al., 2018). This approach has been increasingly adopted in recent years for the study of various horticultural plants, including *Dendranthema grandiflorum*, *Chimonanthus praecox*, and *C. sinensis* (Shang et al., 2020; Wei et al., 2018; Zhao et al., 2018). In this research, the combined analysis of transcriptomes obtained through pooled sequencing and fragrance metabolism data was employed to elucidate the correlation between gene transcription and volatile compounds. This integration of metabolic and gene expression profiles has proven to be a fruitful strategy for identifying candidate genes responsible for the synthesis of fragrance compounds. Similar approaches have been applied in the floral scent analysis of various plants, such as *Freesia hybrid*, tree peony, and *Cananga odorata* (Huang et al., 2018; Jin et al., 2015; Zhang et al., 2021). Within herbaceous peony, a multitude of isoforms were identified, with the majority being associated with monoterpene synthesis. As a result, the investigation of DEGs linked to monoterpene biosynthesis offers valuable insights into the molecular mechanisms underlying the floral scent of herbaceous peony.

In our study, we obtained a comprehensive dataset of 37,878 isoforms and identified 14,369 DEGs through pooled sequencing. By comparing gene expression levels across three distinct floral developmental stages, we identified 2,151 isoforms with significantly different expression levels in the three comparison groups. These DEGs were notably enriched in pathways associated with the biosynthesis of secondary metabolites, with a particular focus on pathways such as “terpenoid backbone biosynthesis” and “monoterpenoid biosynthesis”. This analysis led us to investigate the pathways relevant to terpene biosynthesis, specifically the MVA and MEP pathways (Vranova et al., 2013; Tholl, 2015). The MEP pathway is recognized for its role in monoterpenes production (Tholl, 2015). Based on our findings, the expression patterns of 20 DXS genes were highest in the S3 stage, followed by the S2 stage, and lowest in the S1 stage, mirroring the emission pattern of monoterpenes. However, there were also other DXS genes that exhibited an inverse trend. This phenomenon is not unique to our study, as similar observations have been reported in other floral plants. For example, in *F. hybrid*, Huang et al. (2018) observed that *FhDXS1A* and *FhDXS1B* expression levels decreased during floral development, while *FhDXS2A* increased, highlighting the significant role of *FhDXS2A* in terpene biosynthesis. A comparable pattern was found in *Hedychium coronarium*, where *HcDXS2A* expression was in line with the release of monoterpenes, while *HcDXS1A*, *HcDXS2B*, and *HcDXS3* displayed relatively stable expression levels during flower opening, and *HcDXS1B* expression decreased (Yue et al., 2015). These findings align with the varying expression trends observed in the DXS genes in our study. The regulatory role of DXS genes in terpenoid biosynthesis has been documented in multiple floral plants, including *Lavandula latifolia*, *Withania somnifera*, *Rosa rugosa*, *Catharanthus roseus*, and *Aquilaria sinensis* (Chahed et al., 2000; Munoz-Bertomeu et al., 2006; Feng et al., 2014; Xu et al., 2014; Jadaun et al., 2017). DXS genes are categorized into three distinct clades: DXS I, DXS II, and DXS III. DXS I genes are considered housekeeping genes, encoding enzymes responsible for catalyzing precursor molecules into terpenoids (Kim et al., 2005). DXS II genes are associated with plant-specific secondary metabolites (Walter et al., 2002), while DXS III genes encode enzymes with functions that are either unknown or inactivated (Lichtenthaler, 1999). Research has shown that the ectopic expression of the *AtDXSI* gene in spike lavender resulted in increased essential oil content, particularly enriched in monoterpenes (Munoz-Bertomeu et al., 2006). Additionally, in *H. coronarium*, *HcDXS2A*, belonging to the DXS II clade, is involved in the synthesis of monoterpenes (Yue et al., 2015). Among the twenty up-regulated DXS genes in our study, nineteen belong to the DXS I clade, while one belongs to the DXS II clade. This suggests that these twenty DXS genes likely play a role in the monoterpenoid metabolic pathway involved in the production of fragrance compounds in ‘Wu Hua Long Yu’. In addition to DXS, GPPS and FPPS genes are responsible for producing terpene precursors, namely, GPP and FPP. GPPS primarily governs the synthesis of monoterpenes, while FPPS regulates the biosynthesis of sesquiterpenes (Zhang et al., 2021). Our RNA-seq data demonstrated that the expression patterns of five GPPS and two FPPS genes closely mirrored the release trends of the corresponding volatile compounds. In the context of GPPS, which predominantly provides precursors for monoterpenes biosynthesis (Zhou et al., 2015), we identified ten GPPS genes in our study. Among these, five GPPS genes exhibited up-regulation in the S3 stage compared to the S1 stage, which corresponded with the emission of monoterpenes. GPPS genes can be categorized into homodimeric GPPS and heterodimeric GPPS. Heterodimeric GPPS consists of two subunits: the small regulatory subunit (SSU) and the catalytic LSU. Both homodimeric GPPS and heterodimeric GPPS have been implicated in monoterpene production in various plant species, including *Clarkia breweri*, *Phalaenopsis bellina*, *Humulus lupulus*, and *A. thaliana* (Tholl et al., 2004; Hsiao et al., 2008; Wang and Dixon, 2009; Chen et al., 2015). In our study, four GPPS genes were classified into the homodimeric GPPS clade, while one GPPS belonged to the GPPS-SSU2 clade. This suggests that these five GPPS genes may play a role in the synthesis of monoterpenoids as part of the fragrance production mechanism in ‘Wu Hua Long Yu’.

As the key enzyme in terpene synthesis, TPS genes can be categorized into monoterpene synthases and sesquiterpene synthases based on their products (Tholl, 2006). High expression levels of TPS genes have been identified as the primary reason for the high release of terpenes (Aros et al., 2012; Hu et al., 2016). TPS enzymes have the ability to catalyze various substrates, resulting in different terpene products. For instance, GPP can be converted into monoterpenes by TPS, while FPP can produce sesquiterpenes under the same catalysis (Nagegowda et al., 2008). In *H. coronarium*, the *HcTPS8* gene can catalyze both GPP and FPP to produce monoterpene and sesquiterpene volatiles, respectively (Yue et al., 2014). In our study, we identified thirteen TPS genes associated with the MEP pathway from the DEGs, of which three exhibited expression profiles consistent with the emission of monoterpenes. TPS genes are classified into seven subfamilies: TPS-a, TPS-b, TPS-c, TPS-d, TPS-e/f, TPS-g, and TPS-h (Bohlmann et al., 1998). The subfamilies particularly relevant to monoterpene synthesis are TPS-b and TPS-g. TPS-b genes specifically encode monoterpene synthases featuring the R(R)x8W domain in angiosperms, whereas TPS-g genes encode monoterpene synthases without the R(R)x8W domain (Gao et al., 2018). For instance, in *A. thaliana*, *AtTPS14* belongs to the TPS-g branch and catalyzes the biosynthesis of the monoterpene linalool (Tholl and Lee, 2011). Additionally, *Medicago sativa* contains 24 TPS genes, of which six belong to the TPS-g clade, and they mainly produce monoterpenes (Parker et al., 2014). In our study, the three up-regulated mono-TPS genes were members of the TPS-g clade, and their expression profiles were associated with the release of monoterpenes in ‘Wu Hua Long Yu’.

Considering that monoterpenoids are the predominant volatile compounds in ‘Wu Hua Long Yu’, our transcriptome analysis revealed that more genes were enriched in the MEP pathway compared to the MVA pathway. Some key enzymes within these two pathways exhibited profiles that were consistent with the release trends of the corresponding volatile compounds. This result contributed to the enhanced production of monoterpenoids and the reduction of sesquiterpenoids. DXS, GPPS, and TPS genes are likely to be the key regulators of monoterpenoid release in herbaceous peony. Specifically, GPPS can interact with both upstream DXS and downstream TPS, making it a very important candidate gene.

TFs have been shown to play a critical role in the regulation of secondary metabolite content by binding to the promoters of target genes (Yang et al., 2012). Previous research has demonstrated that TFs can simultaneously regulate the expression of multiple key genes within terpene metabolism-related gene clusters (Zhou et al., 2016). Therefore, identifying key TFs is crucial for advancing the understanding of plant terpenoid synthesis. In our study, we identified ten TFs that may significantly contribute to the synthesis of monoterpenoids, including one NAC, two MYB, three HD-ZIP, one ERF, one C2H2, one HB-other, and one TALE. These TFs have the potential to orchestrate the regulation of monoterpenoid production in ‘Wu Hua Long Yu’. NAC is a plant-specific TF (Olsen et al., 2005). Previous studies have shown that NAC TFs can play essential roles in regulating the accumulation of various secondary metabolites in plants. For example, *AaNAC1* promotes the accumulation of artemisinin (Lv et al., 2016), *SlNAC4* regulates carotenoid accumulation in tomatoes (Zhu et al., 2014), and *AaNAC3* in *Actinidia arguta* interacts with the promoter of monoterpene synthase TPS1, leading to an increase in monoterpene content (Nieuwenhuizen et al., 2015). In this study, *Isoform0029518*, which belongs to the NAC family, exhibits high expression in S3, significantly higher than in S1. This suggests that *Isoform0029518* may serve as an important TF regulating monoterpene synthesis in herbaceous peony. MYB TFs are widely distributed in animals and plants and are known to play a key role in regulating the synthesis of terpenoids (Paz-Ares et al., 1987). Research has shown that *AtMYB21* positively regulates the expression of *AtTPS11*, *AtTPS14*, and *AtTPS21* in *A. thaliana*, increasing the release of terpenoids (Yang et al., 2020b). Similar findings have been reported in other plants, including *Salvia miltiorrhiza*, *Artemisia annua*, and *F. hybrida* (Reeves et al., 2012; Ding et al., 2017; Matías-Hernández et al., 2017; Yang et al., 2020b), all of which are related to terpenoid synthesis. In this study, *Isoform0031453* and *Isoform0030322* are MYB TFs that may be involved in the regulation of terpenoid synthesis. ERF TFs belong to the AP2/ERF family and are unique to plants (Sakuma et al., 2002). Research has indicated that ERF TFs, such as *AaERF1* and *AaERF2* in *A. annua* and *CitERF71* in *Citrus sinensis*, are involved in regulating the synthesis of terpenoids (Yu et al., 2012; Li et al., 2017). In this study, *Isoform0028752*, an ERF TF, may play a significant role in regulating monoterpene synthesis in herbaceous peony. HD-ZIP, C2H2, HB-other, and TALE TFs are known for their functions in plant growth and resistance to environmental stress (Chrispeels et al., 2000; Hamant and Pautot, 2010; Ke et al., 2017; Shen et al., 2018). While there is limited research on the involvement of these TF types in terpenoid synthesis, our study has identified three HD-ZIP, one C2H2, one HB-other, and one TALE TFs that may be related to the synthesis of monoterpenes. However, their functions still require verification. In addition to these TFs with strong correlations, seven additional TFs were identified through the protein-protein interaction (PPI) network, including one NF-YB, one BBX, two C3H, two bHLH, and one CO-like. Among these, *DobHLH4* involved in linalool biosynthesis in *Dendrobium officinale* (Yu et al., 2020). However, the connection between NF-YB, BBX, C3H, CO-like TFs, and floral fragrance has not been extensively studied. The interaction of C3H TFs with both DXS and GPPS proteins suggests that they may serve as important TFs in the synthesis of monoterpenes in ‘Wu Hua Long Yu’. Further research will be needed to confirm their functions.

In summary, our study identified a total of 1193 TFs in the *P. lactiflora* ‘Wu Hua Long Yu’ transcriptome. Among these, 96 TFs were clustered in profile 7, showing a positive correlation with the release of monoterpenes, indicating their potential involvement in monoterpene biosynthesis. Some key TFs were selected for further investigation. These findings contribute to our understanding of the synthetic pathways of plant terpenoids and enrich the knowledge of the regulatory mechanisms of plant secondary metabolism. The DEGs and TFs identified in our study provide valuable candidate genes for future research on floral fragrance in herbaceous peony.

## 5 Conclusions

In our study, we investigated the volatile compounds present in ‘Wu Hua Long Yu’ at five different developmental stages and established that monoterpenoids represent the predominant aroma compounds. We conducted a comprehensive pooled RNA-seq analysis, focusing on three critical developmental periods (S1, S2, and S3) characterized by significant variations in volatile content. This approach allowed us to build a highly accurate transcriptome database. Our analysis revealed a total of 37,878 isoforms and 14,369 DEGs. By combining transcriptome data with volatile analysis using GC-MS, we delved into the molecular mechanisms underpinning the formation of floral scents. As a result, we identified a series of potential candidate genes related to monoterpene biosynthesis. Our findings, based on gene expression profiles, annotations, PPI and correlation analyses, point to DXSs, GPPSs, TPSs, and certain TFs as potential regulators of volatile monoterpenes’ production in ‘Wu Hua Long Yu.’ Notably, many of these genes have pivotal roles in the fragrance biosynthesis of other plant species as well. The extensive data collected from the full-length transcripts of ‘Wu Hua Long Yu’ provide a more detailed understanding of the molecular basis of its fragrance. The candidate genes identified in this study lay the groundwork for future research aimed at breeding fragrant herbaceous peony varieties.

## Data availability statement

The original contributions presented in the study are included in the article/**Supplementary Material**, further inquiries can be directed to the corresponding author. The sequencing data were deposited in the Sequence Read Archive (SRA) database (https://www.ncbi.nlm.nih.gov/sra) with the accession number SRP287892.

## Author contributions

QZ: Investigation, Methodology, Resources, Software, Validation, Writing – original draft. MZ: Investigation, Methodology, Writing – original draft. LG: Investigation, Resources, Writing – review & editing. ZY: Investigation, Resources, Writing – review & editing. YL: Investigation, Resources, Writing – review & editing. JL: Conceptualization, Funding acquisition, Project administration, Supervision, Writing – original draft. YZ: Conceptualization, Data curation, Supervision, Writing – review & editing.
