# Supplementary material for: Transcriptome and volatile compounds analyses of floral development provide insight into floral scent formation in Paeonia lactiflora ‘Wu Hua Long Yu’
Source: Front Plant Sci. 2024 Feb 16;15:1303156. doi: 10.3389/fpls.2024.1303156 (PMC10904628; doi:10.3389/fpls.2024.1303156)
Supplement: Supplementary file 1 [file DataSheet_1.docx]

Supplementary Material

# Supplementary Figures and Tables

## Supplementary Figures


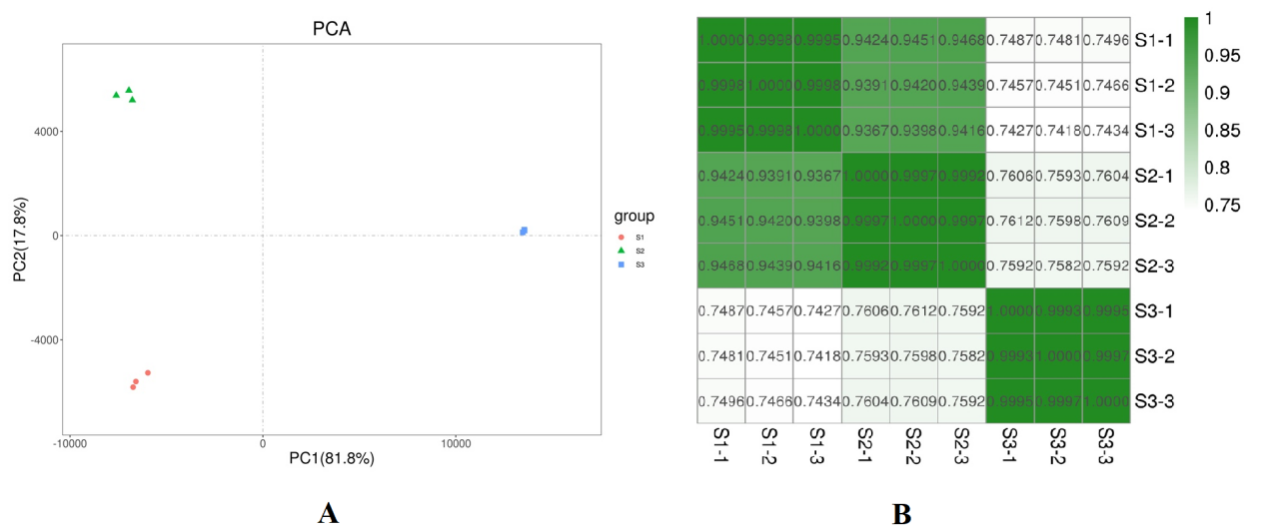


**Supplementary Figure S1.** Summary of the sample clustering. (A) Principal component analysis of the 9 samples based on expression levels. (B) The person correlation between the 9 samples based on expression levels.


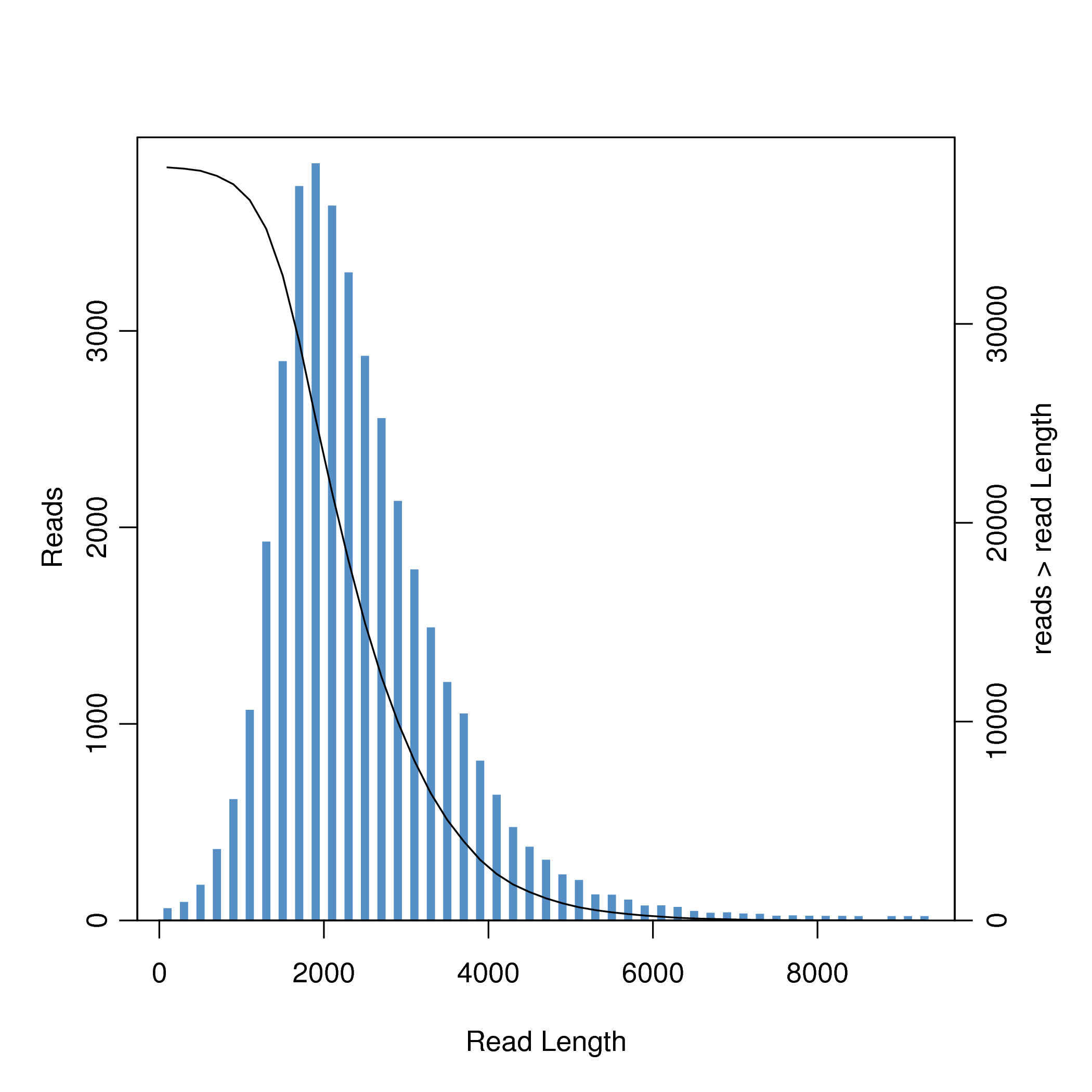


**Supplementary Figure S2.** Length distribution of assembled isoforms.


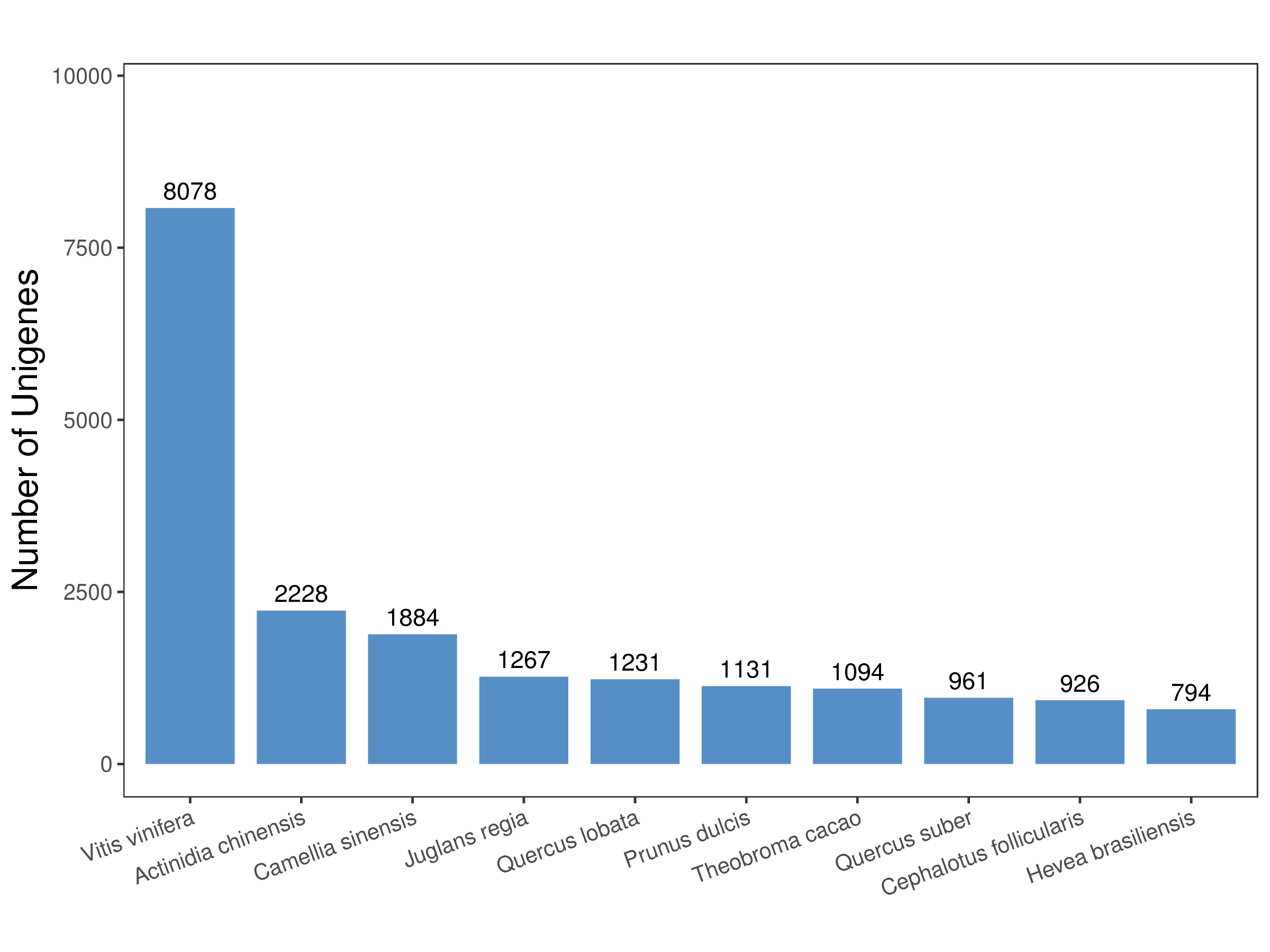


**Supplementary Figure S3.** Species distribution of the top 10 BLAST hits for all homologous sequences.


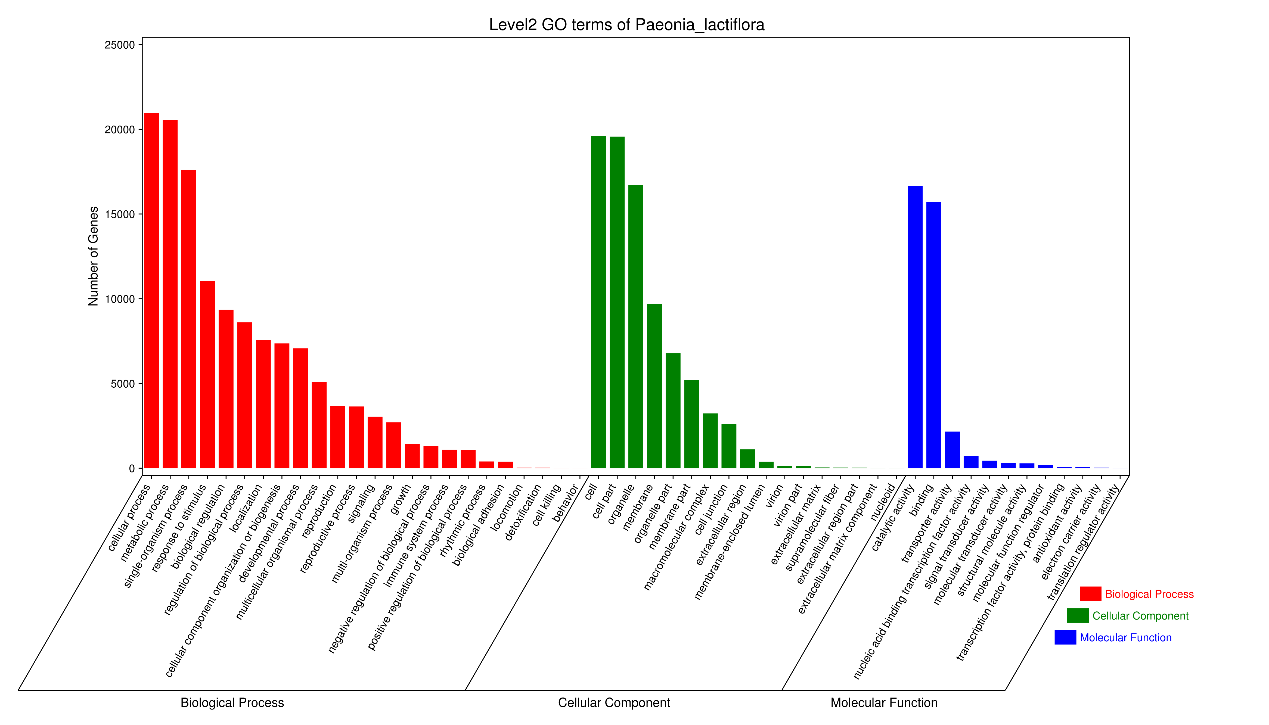


**Supplementary Figure S4.** GO classification of isoforms.


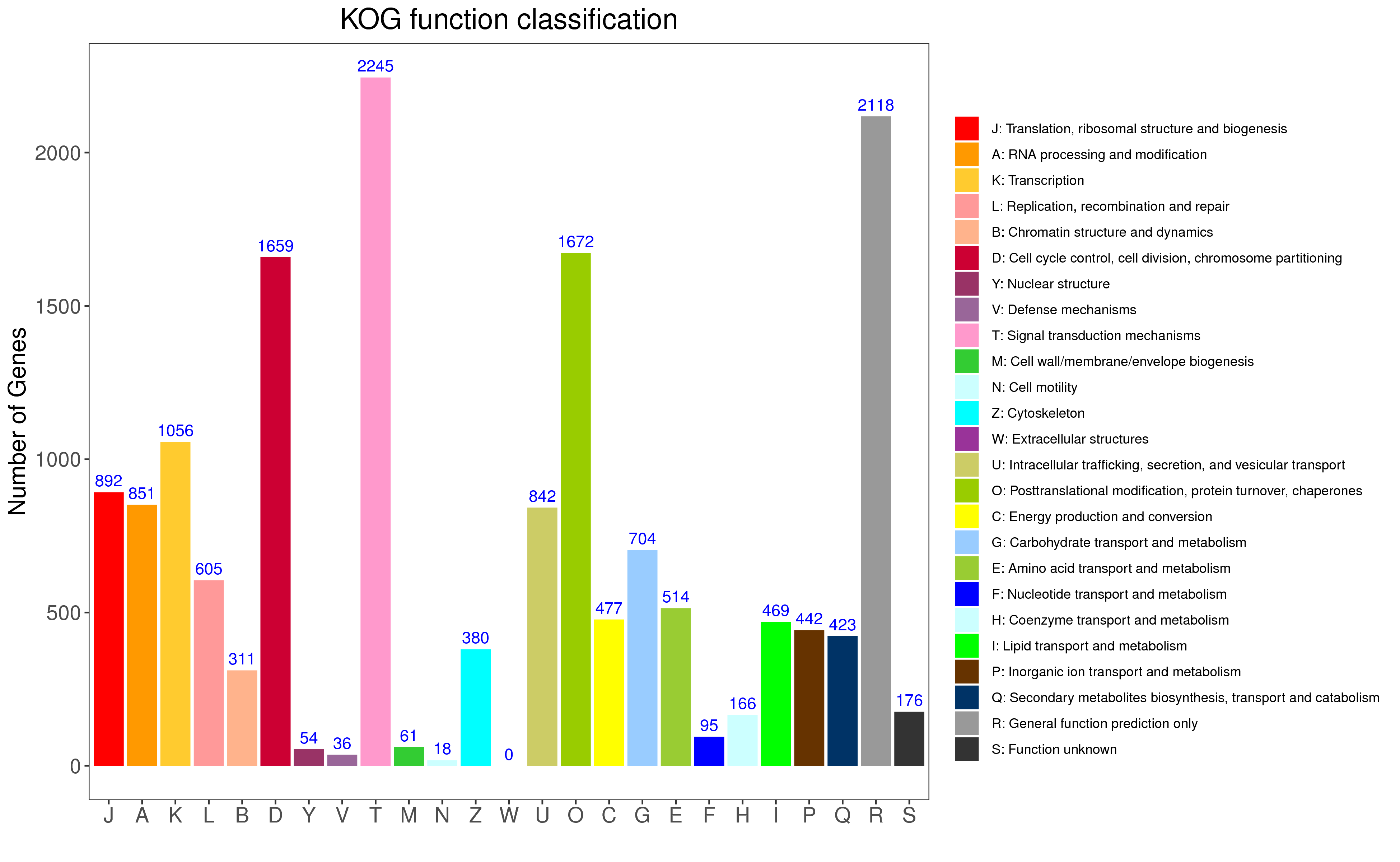
 **Supplementary Figure S5.** KOG functional classification of isoforms.


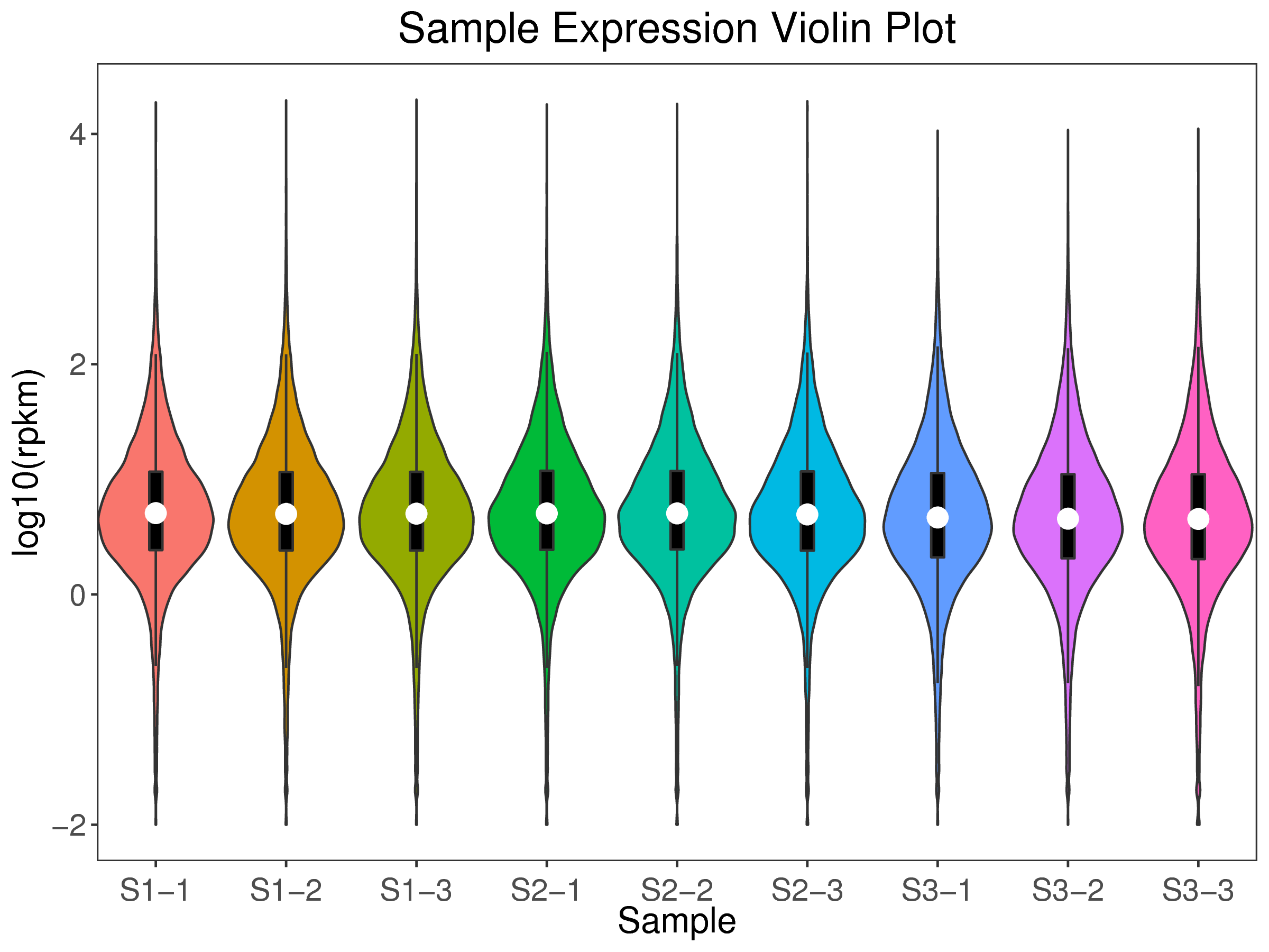


**Supplementary Figure S6.** The violin plot of the gene expression level distribution of each sample.


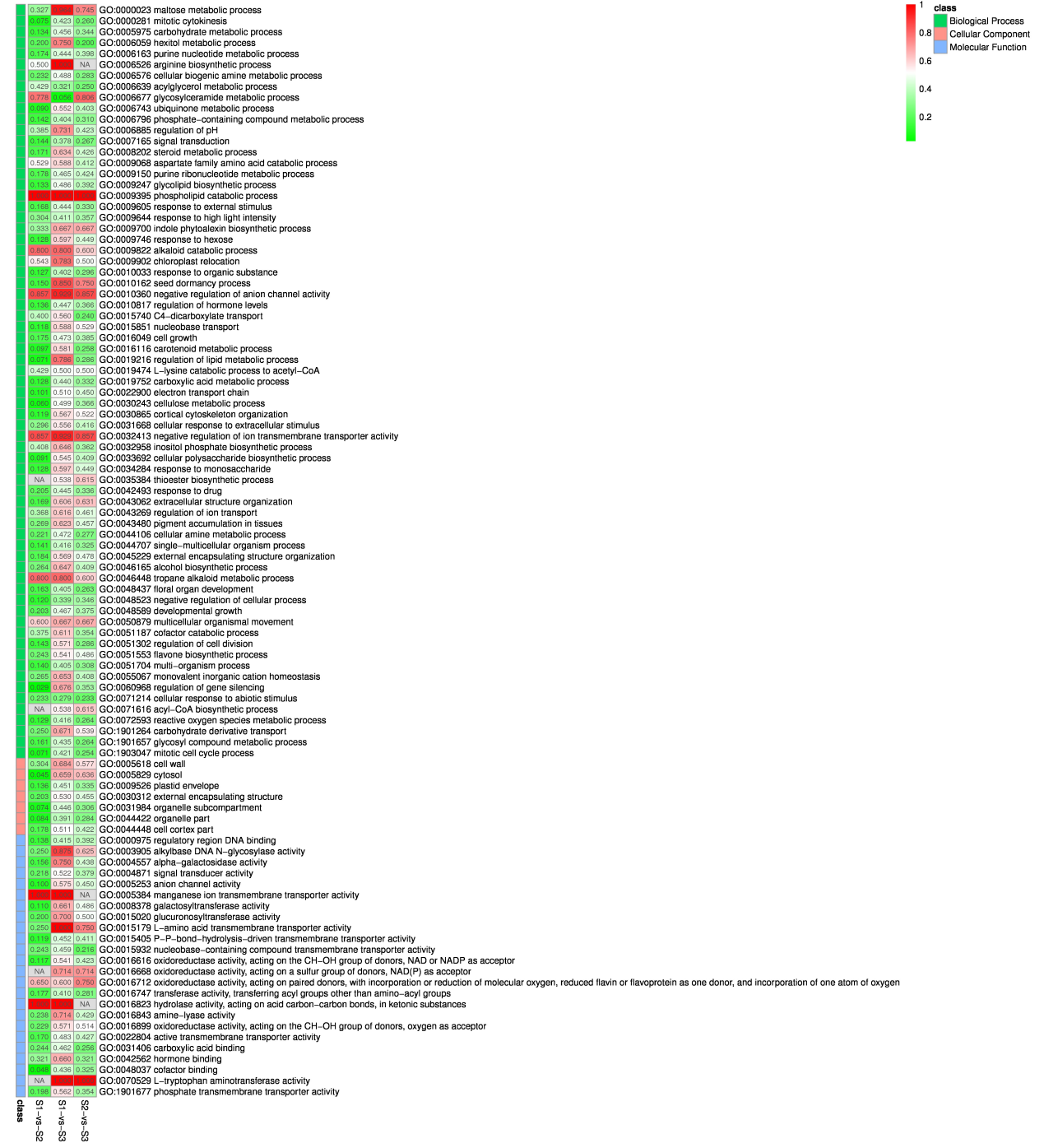


**Supplementary Figure S7.** GO Enrichment factor heatmap of DEGs among three stages.


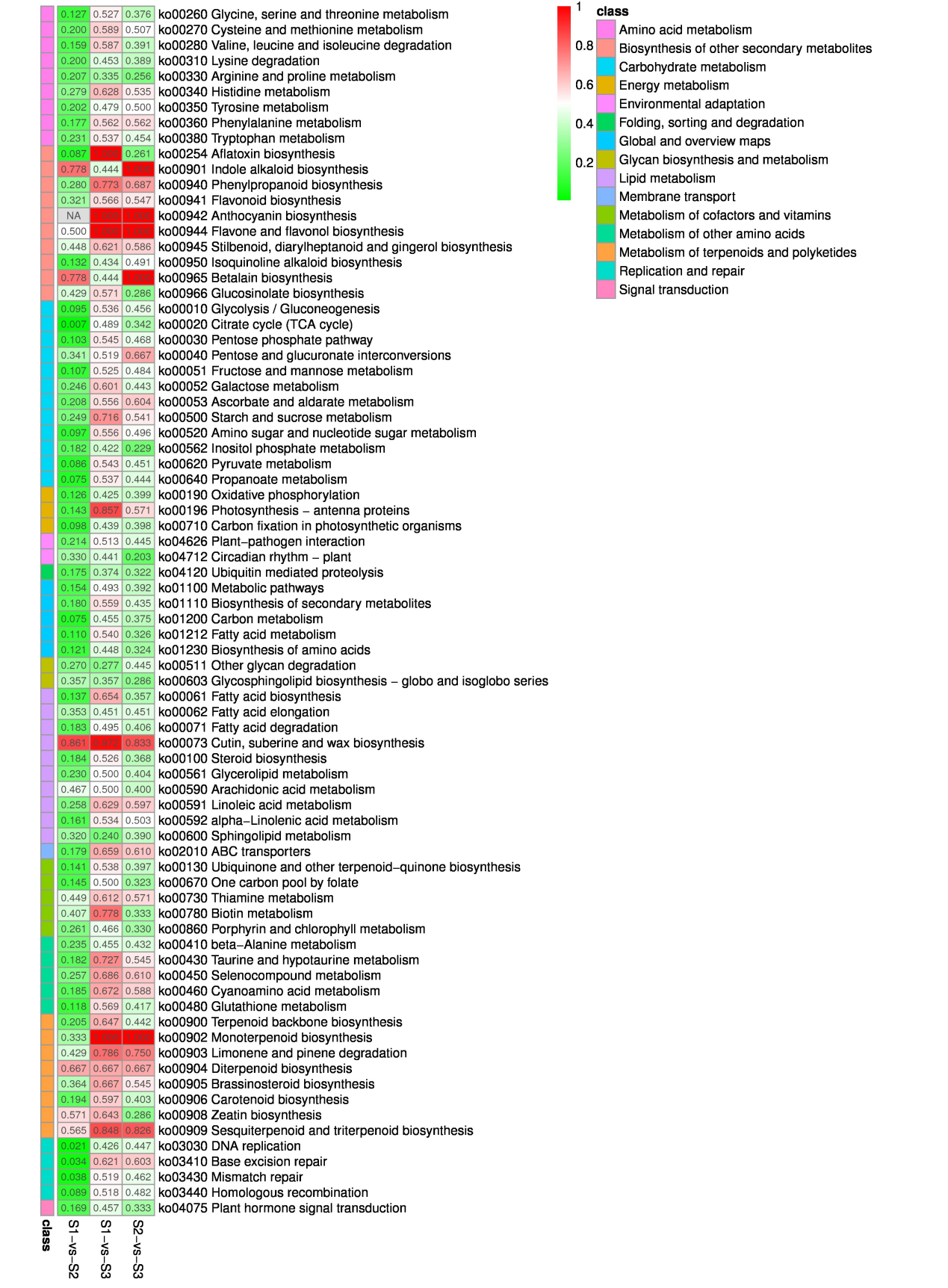


**Supplementary Figure S8.** KEGG Enrichment factor heatmap of DEGs among three stages.


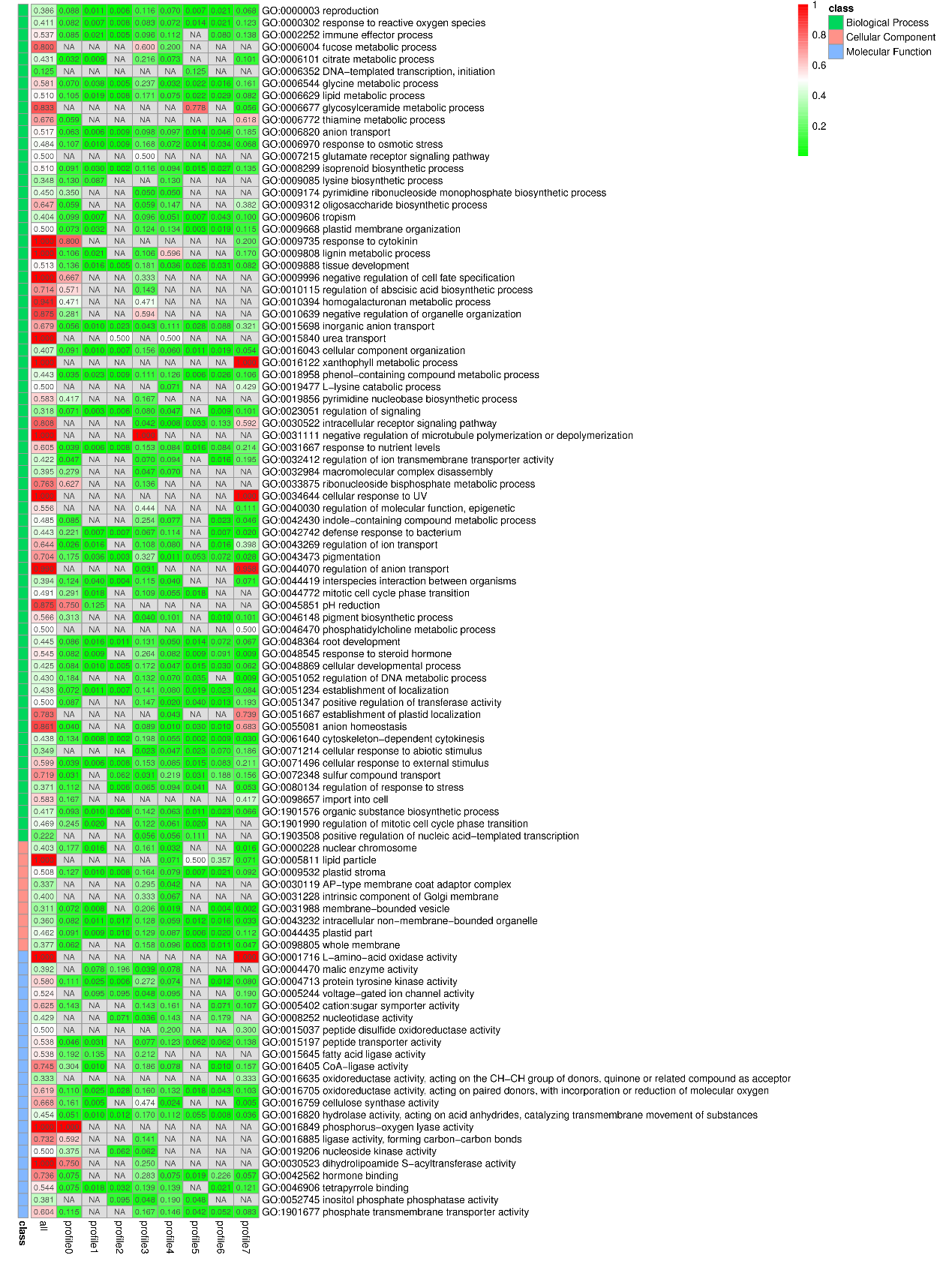


**Supplementary Figure S9.** GO enrichment factor heatmap of genes in profile 0-7.


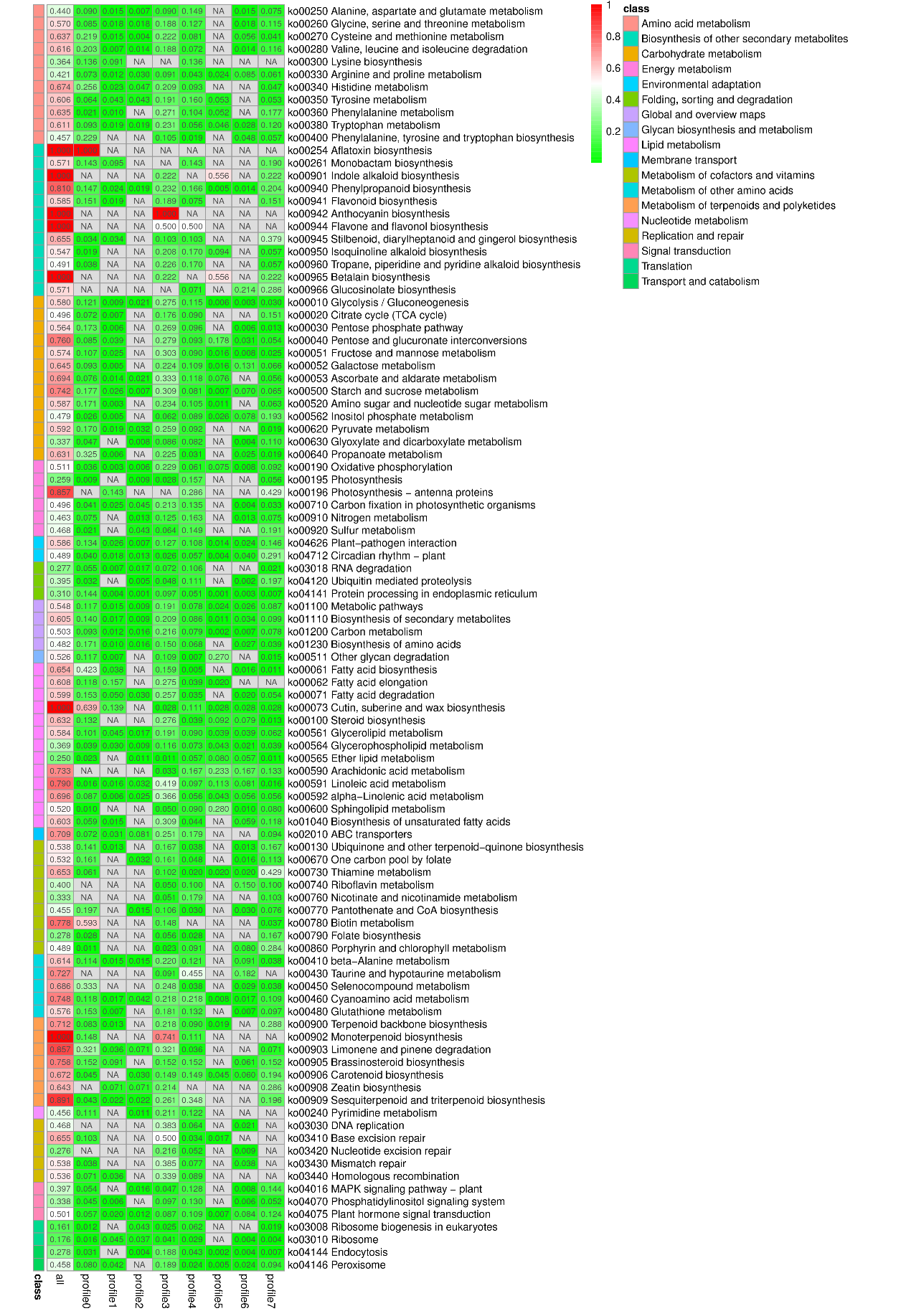


**Supplementary Figure S10.** KEGG enrichment factor heatmap of genes in profile 0-7.

## Supplementary Tables

**Supplementary Table S1.** Sequences of primers used in the qRT-PCR analysis.

| Gene | Gene ID | Forward primer (5’-3’) | Reverse primer (5’-3’) |
| --- | --- | --- | --- |
| *GAPDH* | - | TGTTCACCGACAAAGACAAGG | TAGCCAAGGGAGCAAGACAAT |
| *ARF* | Isoform0008556 | GCCACTGATTTCGTTGCCTC | TGACGGAAGGTTTGGGTAGC |
| *bHLH* | Isoform0031870 | CAAGCCTAACGACAATGGCA | AAGAGGGAGTTGTGAGCGGA |
| *bZIP* | Isoform0027142 | TTTTGCGACTCCCATGCCTT | GCAGGTGATCCAGTCCCAAC |
| *DBB* | Isoform0030505 | GTGATTTGTTGCGCTGACGA | CGAGGGAGCTTGTTGGAGAG |
| *Dof* | Isoform0020517 | GATACTTCCTTGCCCACGCT | AGCTCCCACTGGCACATTAC |
| *ERF* | Isoform0028752 | TGGGTTTTGGAGATTCGGCA | CCTCTCAATTCCCCCGCTAC |
| *GRAS* | Isoform0018196 | CGGCGCTTAAATCACGACTG | GTGTTGGCGTCCGATGTTTC |
| *GRAS* | Isoform0012897 | CTGCCCCATTCTTTCCTCGT | GTGACGTTCCACTCTCTCCG |
| *HB* | Isoform0028150 | TGAGTTCAGGTAGCGGTGGA | CCCGTCATCCTCTTCCGATT |
| *MYB* | Isoform0031453 | TCGAAACTCCTCAACGGTCG | TAAAACAGGGACCCGGCAAA |
| *NAC* | Isoform0019267 | GAGCTTTTGCTTTGTGCCGT | GCCTGTAGGATTGCTCTCCA |
| *NF-YB* | Isoform0031505 | TCTTTTGTGGGCGATGACGA | TCTACCCACGGACGTCTTCT |
| *TALE* | Isoform0003876 | CCTGGCTACGTCGGTTACTC | GAAGTTTGTGCCGGAAGTGG |
| *TALE* | Isoform0004670 | TCCACCAGAGGTTTTCAGGC | CGCTCGCCCATTTCTACTCT |
| *TCP* | Isoform0025735 | GGAAGGCGATTTCCCCACAA | TCCAAAAGCACGCGAAACAC |
| *WRKY* | Isoform0015025 | CTTCAGCCCCATTCCCAACT | GCGGGTAAATTTTGTGGCGA |

**Supplementary Table S2.** The volatile compounds identified from GC-MS.

| Time | Compound name | Cas | Molecular formula | Molecular formula | Hit Spectrum and compound structure |
| --- | --- | --- | --- | --- | --- |
| 5.29 | Hexanal | 66-25-1 | C6H12O | 100 |  |
| 6.81 | 2-Hexenal | 505-57-7 | C6H10O | 98 |  |
| 6.88 | 3-Hexen-1-ol | 544-12-7 | C6H12O | 100 |  |
| 7.31 | 1-Hexanol | 111-27-3 | C6H14O | 102 |  |
| 12.26 | 1-Hexanol, 2-ethyl- | 104-76-7 | C8H18O | 130 |  |
| 16.20 | trans-2-Nonenal | NA | C9H16O | 140 |  |
| 17.32 | Dodecane | 112-40-3 | C12H26 | 170 |  |
| 10.11 | Benzaldehyde | 100-52-7 | C7H6O | 106 |  |
| 12.44 | Benzyl alcohol | 100-51-6 | C7H8O | 108 |  |
| 12.71 | Benzaldehyde, 2-hydroxy- | 90-02-8 | C7H6O2 | 122 |  |
| 14.86 | Phenylethyl alcohol | 60-12-8 | C8H10O | 122 |  |
| 16.37 | Benzene, 1,4-dimethoxy- | 150-78-7 | C8H10O2 | 138 |  |
| 14.42 | Linalool | 78-70-6 | C10H18O | 154 |  |
| 16.94 | Myrtanal | 4764-14-1 | C10H16O | 152 |  |
| 18.21 | Citronellol | 106-22-9 | C10H20O | 156 |  |
| 18.92 | Geraniol | 106-24-1 | C10H18O | 154 |  |
| 19.39 | Citral | 5392-40-5 | C10H16O | 152 |  |
| 23.42 | Caryophyllene | 87-44-5 | C15H24 | 204 |  |

**Supplementary Table S3.** Overview of the Illumina HiSeq data.

| Sample | Raw data (bp) | Clean data (bp) | Q30 (%) | GC (%) |
| --- | --- | --- | --- | --- |
| S1-1 | 6,665,272,500 | 6,632,785,500 | 6,169,747,788 (93.02%) | 2,983,706,671 (44.98%) |
| S1-2 | 7,590,911,100 | 7,554,647,959 | 7,069,351,885 (93.58%) | 3,409,485,230 (45.13%) |
| S1-3 | 6,929,917,800 | 6,898,410,852 | 6,405,745,566 (92.86%) | 3,103,969,219 (45.00%) |
| S2-1 | 7,570,245,600 | 7,533,241,829 | 7,019,488,015 (93.18%) | 3,381,698,380 (44.89%) |
| S2-2 | 7,392,323,700 | 7,361,933,510 | 6,813,112,882 (92.55%) | 3,307,990,134 (44.93%) |
| S2-3 | 8,319,895,800 | 8,275,477,869 | 7,630,569,079 (92.21%) | 3,738,288,606 (45.17%) |
| S3-1 | 6,460,895,700 | 6,430,506,849 | 5,996,344,555 (93.25%) | 2,886,336,224 (44.89%) |
| S3-2 | 6,062,033,400 | 6,033,628,257 | 5,615,076,592 (93.06%) | 2,727,187,587 (45.20%) |
| S3-3 | 8,788,601,700 | 8,758,949,896 | 8,136,982,124 (92.90%) | 3,954,029,525 (45.14%) |

**Supplementary Table S4.** Overview of the Pacbio ISO-seq data.

| Sample ID | Total base (bp) | Subreads number | CCS reads  number | Consensus reads number |
| --- | --- | --- | --- | --- |
| SYHB | 50.68 G | 21,796,952 | 665,658 | 48,796 |

**Supplementary Table S5.** Overview of the isoforms obtained from the pooled sequencing.

| Total number | Total length (bp) | Maximum length (bp) | Minimum length (bp) | Average length (bp) | N50 length (bp) | GC content |
| --- | --- | --- | --- | --- | --- | --- |
| 37,878 | 91,871,183 | 9395 | 55 | 2425.45 | 2637 | 42.21% |

**Supplementary Table S6.** Gene numbers in 137 KEGG pathways.

| Pathway | Gene number | Pathway ID |
| --- | --- | --- |
| Metabolic pathways | 5099 | ko01100 |
| Biosynthesis of secondary metabolites | 2965 | ko01110 |
| Carbon metabolism | 888 | ko01200 |
| Biosynthesis of amino acids | 672 | ko01230 |
| Protein processing in endoplasmic reticulum | 667 | ko04141 |
| Starch and sucrose metabolism | 582 | ko00500 |
| Spliceosome | 561 | ko03040 |
| Endocytosis | 554 | ko04144 |
| RNA transport | 527 | ko03013 |
| Ubiquitin mediated proteolysis | 441 | ko04120 |
| Plant-pathogen interaction | 425 | ko04626 |
| Plant hormone signal transduction | 403 | ko04075 |
| Pyruvate metabolism | 370 | ko00620 |
| Oxidative phosphorylation | 358 | ko00190 |
| mRNA surveillance pathway | 358 | ko03015 |
| Amino sugar and nucleotide sugar metabolism | 351 | ko00520 |
| Glycolysis / Gluconeogenesis | 338 | ko00010 |
| RNA degradation | 292 | ko03018 |
| Fatty acid metabolism | 291 | ko01212 |
| Citrate cycle (TCA cycle) | 278 | ko00020 |
| Cysteine and methionine metabolism | 270 | ko00270 |
| MAPK signaling pathway - plant | 257 | ko04016 |
| Glyoxylate and dicarboxylate metabolism | 255 | ko00630 |
| Carbon fixation in photosynthetic organisms | 244 | ko00710 |
| Ribosome | 244 | ko03010 |
| Glycerophospholipid metabolism | 233 | ko00564 |
| Circadian rhythm - plant | 227 | ko04712 |
| ABC transporters | 223 | ko02010 |
| Aminoacyl-tRNA biosynthesis | 218 | ko00970 |
| Peroxisome | 212 | ko04146 |
| Phenylpropanoid biosynthesis | 211 | ko00940 |
| Fatty acid degradation | 202 | ko00071 |
| Inositol phosphate metabolism | 192 | ko00562 |
| Galactose metabolism | 183 | ko00052 |
| Fatty acid biosynthesis | 182 | ko00061 |
| 2-Oxocarboxylic acid metabolism | 181 | ko01210 |
| Glycerolipid metabolism | 178 | ko00561 |
| Purine metabolism | 175 | ko00230 |
| Phagosome | 171 | ko04145 |
| Glycine, serine and threonine metabolism | 165 | ko00260 |
| Arginine and proline metabolism | 164 | ko00330 |
| alpha-Linolenic acid metabolism | 161 | ko00592 |
| Ribosome biogenesis in eukaryotes | 161 | ko03008 |
| Propanoate metabolism | 160 | ko00640 |
| Pentose phosphate pathway | 156 | ko00030 |
| Terpenoid backbone biosynthesis | 156 | ko00900 |
| Phosphatidylinositol signaling system | 154 | ko04070 |
| Ascorbate and aldarate metabolism | 144 | ko00053 |
| Glutathione metabolism | 144 | ko00480 |
| Valine, leucine and isoleucine degradation | 138 | ko00280 |
| Other glycan degradation | 137 | ko00511 |
| Alanine, aspartate and glutamate metabolism | 134 | ko00250 |
| beta-Alanine metabolism | 132 | ko00410 |
| RNA polymerase | 132 | ko03020 |
| Pentose and glucuronate interconversions | 129 | ko00040 |
| Proteasome | 129 | ko03050 |
| Autophagy - other eukaryotes | 125 | ko04136 |
| Fructose and mannose metabolism | 122 | ko00051 |
| Cyanoamino acid metabolism | 119 | ko00460 |
| Nucleotide excision repair | 116 | ko03420 |
| Photosynthesis | 108 | ko00195 |
| Tryptophan metabolism | 108 | ko00380 |
| Phenylalanine, tyrosine and tryptophan biosynthesis | 105 | ko00400 |
| Selenocompound metabolism | 105 | ko00450 |
| Sphingolipid metabolism | 100 | ko00600 |
| Phenylalanine metabolism | 96 | ko00360 |
| Lysine degradation | 95 | ko00310 |
| Tyrosine metabolism | 94 | ko00350 |
| N-Glycan biosynthesis | 93 | ko00510 |
| Protein export | 93 | ko03060 |
| Pyrimidine metabolism | 90 | ko00240 |
| Ether lipid metabolism | 88 | ko00565 |
| Porphyrin and chlorophyll metabolism | 88 | ko00860 |
| Nitrogen metabolism | 80 | ko00910 |
| Ubiquinone and other terpenoid-quinone biosynthesis | 78 | ko00130 |
| Basal transcription factors | 78 | ko03022 |
| Steroid biosynthesis | 76 | ko00100 |
| Arginine biosynthesis | 76 | ko00220 |
| Biosynthesis of unsaturated fatty acids | 68 | ko01040 |
| Carotenoid biosynthesis | 67 | ko00906 |
| Pantothenate and CoA biosynthesis | 66 | ko00770 |
| Various types of N-glycan biosynthesis | 65 | ko00513 |
| Linoleic acid metabolism | 62 | ko00591 |
| One carbon pool by folate | 62 | ko00670 |
| Base excision repair | 58 | ko03410 |
| Butanoate metabolism | 56 | ko00650 |
| Homologous recombination | 56 | ko03440 |
| Flavonoid biosynthesis | 53 | ko00941 |
| Isoquinoline alkaloid biosynthesis | 53 | ko00950 |
| Tropane, piperidine and pyridine alkaloid biosynthesis | 53 | ko00960 |
| Other types of O-glycan biosynthesis | 52 | ko00514 |
| Mismatch repair | 52 | ko03430 |
| SNARE interactions in vesicular transport | 52 | ko04130 |
| Fatty acid elongation | 51 | ko00062 |
| Valine, leucine and isoleucine biosynthesis | 49 | ko00290 |
| Thiamine metabolism | 49 | ko00730 |
| Sulfur metabolism | 47 | ko00920 |
| DNA replication | 47 | ko03030 |
| Sesquiterpenoid and triterpenoid biosynthesis | 46 | ko00909 |
| Histidine metabolism | 43 | ko00340 |
| Glycosylphosphatidylinositol (GPI)-anchor biosynthesis | 41 | ko00563 |
| Nicotinate and nicotinamide metabolism | 39 | ko00760 |
| Cutin, suberine and wax biosynthesis | 36 | ko00073 |
| Folate biosynthesis | 36 | ko00790 |
| Brassinosteroid biosynthesis | 33 | ko00905 |
| Arachidonic acid metabolism | 30 | ko00590 |
| Stilbenoid, diarylheptanoid and gingerol biosynthesis | 29 | ko00945 |
| Limonene and pinene degradation | 28 | ko00903 |
| Biotin metabolism | 27 | ko00780 |
| Monoterpenoid biosynthesis | 27 | ko00902 |
| Glycosaminoglycan degradation | 26 | ko00531 |
| Aflatoxin biosynthesis | 23 | ko00254 |
| Lysine biosynthesis | 22 | ko00300 |
| Monobactam biosynthesis | 21 | ko00261 |
| Synthesis and degradation of ketone bodies | 20 | ko00072 |
| Riboflavin metabolism | 20 | ko00740 |
| C5-Branched dibasic acid metabolism | 18 | ko00660 |
| Vitamin B6 metabolism | 18 | ko00750 |
| Non-homologous end-joining | 16 | ko03450 |
| Phosphonate and phosphinate metabolism | 14 | ko00440 |
| Glycosphingolipid biosynthesis - globo and isoglobo series | 14 | ko00603 |
| Glycosphingolipid biosynthesis - ganglio series | 14 | ko00604 |
| Zeatin biosynthesis | 14 | ko00908 |
| Glucosinolate biosynthesis | 14 | ko00966 |
| Taurine and hypotaurine metabolism | 11 | ko00430 |
| Caffeine metabolism | 10 | ko00232 |
| Indole alkaloid biosynthesis | 9 | ko00901 |
| Betalain biosynthesis | 9 | ko00965 |
| Photosynthesis - antenna proteins | 7 | ko00196 |
| Anthocyanin biosynthesis | 7 | ko00942 |
| Sulfur relay system | 7 | ko04122 |
| Flavone and flavonol biosynthesis | 6 | ko00944 |
| Lipoic acid metabolism | 3 | ko00785 |
| Diterpenoid biosynthesis | 3 | ko00904 |
| Isoflavonoid biosynthesis | 2 | ko00943 |
| Polyketide sugar unit biosynthesis | 1 | ko00523 |
| Glycosphingolipid biosynthesis - lacto and neolacto series | 1 | ko00601 |

**Supplementary Table S7.** The number of sequenced genes in the nine databases.

| Sample | Total genes | Sequenced genes/Total genes (%) |
| --- | --- | --- |
| All | 37,878 | 37,785 (99.75%) |
| S1-1 | 37,878 | 37,380 (98.69%) |
| S1-2 | 37,878 | 37,369 (98.66%) |
| S1-3 | 37,878 | 37,390 (98.71%) |
| S2-1 | 37,878 | 37,369 (98.66%) |
| S2-2 | 37,878 | 37,370 (98.66%) |
| S2-3 | 37,878 | 37,360 (98.63%) |
| S3-1 | 37,878 | 37,236 (98.31%) |
| S3-2 | 37,878 | 37,224 (98.27%) |
| S3-3 | 37,878 | 37,301 (98.48%) |

**Supplementary Table S8.** The reads of total mapped in the nine databases.

| Sample | Total | Unique mapped (%) | Unmapped (%) | Multiple mapped (%) | Total mapped (%) |
| --- | --- | --- | --- | --- | --- |
| S1-1 | 44,377,830 | 7,838,702 (17.66%) | 6,199,301 (13.97%) | 30,339,827 (68.37%) | 38,178,529 (86.03%) |
| S1-2 | 50,548,106 | 8,920,496 (17.65%) | 6,743,067 (13.34%) | 34,884,543 (69.01%) | 43,805,039 (86.66%) |
| S1-3 | 46,136,970 | 8,180,061 (17.73%) | 6,255,228 (13.56%) | 31,701,681 (68.71%) | 39,881,742 (86.44%) |
| S2-1 | 50,391,406 | 8,489,057 (16.85%) | 6,547,274 (12.99%) | 35,355,075 (70.16%) | 43,844,132 (87.01%) |
| S2-2 | 49,199,808 | 8,234,597 (16.74%) | 6,391,869 (12.99%) | 34,573,342 (70.27%) | 42,807,939 (87.01%) |
| S2-3 | 55,375,670 | 9,220,106 (16.65%) | 6,799,338 (12.28%) | 39,356,226 (71.07%) | 48,576,332 (87.72%) |
| S3-1 | 43,020,966 | 7,328,841 (17.04%) | 5,876,918 (13.66%) | 29,815,207 (69.30%) | 37,144,048 (86.34%) |
| S3-2 | 40,369,880 | 6,818,647 (16.89%) | 5,288,901 (13.10%) | 28,262,332 (70.01%) | 35,080,979 (86.90%) |
| S3-3 | 58,518,790 | 9,971,366 (17.04%) | 7,742,635 (13.23%) | 40,804,789 (69.73%) | 50,776,155 (86.77%) |

**Supplementary Table S9.** The genes involved in monoterpene synthesis.

| Gene ID | Description | S1-mean | S2-mean | S3-mean |
| --- | --- | --- | --- | --- |
| Isoform0005370 | DXS | 0.09 | 1.40 | 5.31 |
| Isoform0005646 | DXS | 0.57 | 5.26 | 22.80 |
| Isoform0005699 | DXS | 3.36 | 27.70 | 117.32 |
| Isoform0006454 | DXS | 0.64 | 5.65 | 24.40 |
| Isoform0006850 | DXS | 17.51 | 18.20 | 13.12 |
| Isoform0007194 | DXS | 1.93 | 17.56 | 73.18 |
| Isoform0007808 | DXS | 11.84 | 9.45 | 0.04 |
| Isoform0008059 | DXS | 1.52 | 0.93 | 1.43 |
| Isoform0008191 | DXS | 2.31 | 19.39 | 81.79 |
| Isoform0009642 | DXS | 12.76 | 9.92 | 0.07 |
| Isoform0010604 | DXS | 2.48 | 18.93 | 83.13 |
| Isoform0011871 | DXS | 2.96 | 29.11 | 124.17 |
| Isoform0013423 | DXS | 3.43 | 31.06 | 133.73 |
| Isoform0014719 | DXS | 3.12 | 26.72 | 113.33 |
| Isoform0015022 | DXS | 9.20 | 8.03 | 0.02 |
| Isoform0017217 | DXS | 0.56 | 4.31 | 18.50 |
| Isoform0018118 | DXS | 2.73 | 21.54 | 94.17 |
| Isoform0020550 | DXS | 1.81 | 18.40 | 80.84 |
| Isoform0021612 | DXS | 4.25 | 3.29 | 0.03 |
| Isoform0022923 | DXS | 2.38 | 21.05 | 86.67 |
| Isoform0023122 | DXS | 1.73 | 15.26 | 66.78 |
| Isoform0024133 | DXS | 2.93 | 10.73 | 4.13 |
| Isoform0025186 | DXS | 1.30 | 3.46 | 12.57 |
| Isoform0025353 | DXS | 2.08 | 16.76 | 68.06 |
| Isoform0027642 | DXS | 1.84 | 16.02 | 66.28 |
| Isoform0029168 | DXS | 1.83 | 16.68 | 68.02 |
| Isoform0035564 | DXS | 1.03 | 8.73 | 35.80 |
| Isoform0016237 | DXR | 55.02 | 73.60 | 27.55 |
| Isoform0018511 | DXR | 43.21 | 59.27 | 22.30 |
| Isoform0021059 | DXR | 58.71 | 79.71 | 29.20 |
| Isoform0022601 | DXR | 31.76 | 43.60 | 16.01 |
| Isoform0023863 | DXR | 6.43 | 7.21 | 3.50 |
| Isoform0027584 | DXR | 41.98 | 57.16 | 20.46 |
| Isoform0030875 | DXR | 23.55 | 32.30 | 12.13 |
| Isoform0032944 | DXR | 11.81 | 15.70 | 5.04 |
| Isoform0033030 | DXR | 0.33 | 0.20 | 0.04 |
| Isoform0031172 | MCT | 29.26 | 18.63 | 14.49 |
| Isoform0026855 | CMK | 26.80 | 18.55 | 1.62 |
| Isoform0028126 | CMK | 97.51 | 68.02 | 5.86 |
| Isoform0032368 | MDS | 201.26 | 70.94 | 26.09 |
| Isoform0023871 | MDS | 18.06 | 6.51 | 2.28 |
| Isoform0004050 | HDS | 16.47 | 14.19 | 7.66 |
| Isoform0004302 | HDS | 16.78 | 15.22 | 8.62 |
| Isoform0004457 | HDS | 17.10 | 15.59 | 8.35 |
| Isoform0004766 | HDS | 16.37 | 13.87 | 7.95 |
| Isoform0005219 | HDS | 20.47 | 17.54 | 9.34 |
| Isoform0005254 | HDS | 15.99 | 13.23 | 7.50 |
| Isoform0005306 | HDS | 14.90 | 12.25 | 7.06 |
| Isoform0005320 | HDS | 18.38 | 15.84 | 8.52 |
| Isoform0005524 | HDS | 14.97 | 12.54 | 7.24 |
| Isoform0005830 | HDS | 18.50 | 16.62 | 9.62 |
| Isoform0007986 | HDS | 10.63 | 8.63 | 5.28 |
| Isoform0010395 | HDS | 7.43 | 6.91 | 3.71 |
| Isoform0011109 | HDS | 11.07 | 10.15 | 5.71 |
| Isoform0012659 | HDS | 15.68 | 14.01 | 8.10 |
| Isoform0014759 | HDS | 16.52 | 14.15 | 7.78 |
| Isoform0016156 | HDS | 14.48 | 12.51 | 6.80 |
| Isoform0017871 | HDS | 13.43 | 11.34 | 5.86 |
| Isoform0024174 | HDS | 9.28 | 9.11 | 4.66 |
| Isoform0025476 | HDS | 12.30 | 10.45 | 5.81 |
| Isoform0028832 | HDS | 9.99 | 8.39 | 5.28 |
| Isoform0030485 | HDS | 12.20 | 10.38 | 6.07 |
| Isoform0030314 | HDR | 67.00 | 62.47 | 48.66 |
| Isoform0022444 | HDR | 138.43 | 133.31 | 99.07 |
| Isoform0024310 | HDR | 170.32 | 165.00 | 126.64 |
| Isoform0029258 | HDR | 76.35 | 76.19 | 57.38 |
| Isoform0022374 | GPPS | 7.67 | 13.39 | 20.41 |
| Isoform0022826 | GPPS | 9.12 | 14.20 | 21.37 |
| Isoform0024241 | GPPS | 22.46 | 16.55 | 9.96 |
| Isoform0025417 | GPPS | 3.68 | 7.18 | 9.51 |
| Isoform0027065 | GPPS | 24.89 | 16.98 | 10.27 |
| Isoform0027817 | GPPS | 40.65 | 31.58 | 17.94 |
| Isoform0027912 | GPPS | 5.31 | 9.02 | 23.83 |
| Isoform0029797 | GPPS | 10.69 | 10.51 | 18.56 |
| Isoform0031454 | GPPS | 318.05 | 1177.04 | 567.75 |
| Isoform0033073 | GPPS | 5.83 | 27.60 | 12.28 |
| Isoform0006186 | TPS | 20.92 | 43.30 | 0.16 |
| Isoform0013736 | TPS | 12.71 | 3.86 | 0.77 |
| Isoform0014930 | TPS | 7.52 | 137.87 | 76.76 |
| Isoform0016842 | TPS | 6.90 | 121.22 | 70.80 |
| Isoform0018347 | TPS | 0.01 | 0.18 | 2.76 |
| Isoform0019028 | TPS | 26.34 | 7.36 | 1.39 |
| Isoform0019388 | TPS | 38.43 | 80.74 | 0.35 |
| Isoform0019653 | TPS | 0.13 | 0.17 | 187.38 |
| Isoform0021322 | TPS | 0.21 | 0.69 | 20.04 |
| Isoform0025075 | TPS | 28.05 | 62.82 | 0.24 |
| Isoform0026623 | TPS | 14.34 | 4.52 | 0.83 |
| Isoform0027933 | TPS | 1.11 | 20.94 | 10.61 |
| Isoform0028441 | TPS | 26.54 | 61.19 | 0.40 |

**Supplementary Table S10.** The genes involved in sesquiterpene synthesis.

| Gene ID | Description | S1-mean | S2-mean | S3-mean |
| --- | --- | --- | --- | --- |
| Isoform0022786 | AACT | 9.03 | 10.20 | 8.76 |
| Isoform0023742 | AACT | 5.54 | 6.44 | 11.75 |
| Isoform0024694 | AACT | 11.07 | 12.15 | 21.36 |
| Isoform0024709 | AACT | 10.09 | 11.29 | 9.08 |
| Isoform0025313 | AACT | 7.18 | 8.65 | 14.25 |
| Isoform0026643 | AACT | 50.76 | 30.22 | 29.08 |
| Isoform0026723 | AACT | 106.95 | 62.29 | 57.49 |
| Isoform0027570 | AACT | 7.83 | 8.68 | 14.08 |
| Isoform0027581 | AACT | 5.02 | 5.08 | 8.63 |
| Isoform0021906 | HMGS | 38.14 | 23.14 | 39.67 |
| Isoform0022660 | HMGS | 24.96 | 18.33 | 10.15 |
| Isoform0024235 | HMGS | 42.42 | 28.36 | 10.20 |
| Isoform0003095 | HMGR | 7.29 | 12.06 | 20.41 |
| Isoform0004275 | HMGR | 8.44 | 13.18 | 24.76 |
| Isoform0007886 | HMGR | 3.92 | 6.93 | 12.79 |
| Isoform0009580 | HMGR | 0.89 | 1.22 | 43.67 |
| Isoform0010734 | HMGR | 6.51 | 7.94 | 23.64 |
| Isoform0011629 | HMGR | 3.83 | 4.63 | 35.47 |
| Isoform0011717 | HMGR | 15.15 | 24.99 | 39.81 |
| Isoform0012164 | HMGR | 5.88 | 10.17 | 18.04 |
| Isoform0013042 | HMGR | 1.61 | 0.97 | 3.47 |
| Isoform0013458 | HMGR | 10.02 | 15.42 | 28.17 |
| Isoform0013501 | HMGR | 1.84 | 1.90 | 59.34 |
| Isoform0014036 | HMGR | 10.16 | 17.52 | 28.30 |
| Isoform0015024 | HMGR | 21.28 | 3.30 | 2.40 |
| Isoform0016171 | HMGR | 8.80 | 14.07 | 22.76 |
| Isoform0016494 | HMGR | 16.17 | 17.94 | 55.94 |
| Isoform0016633 | HMGR | 11.52 | 18.72 | 30.40 |
| Isoform0021689 | HMGR | 7.83 | 14.37 | 25.93 |
| Isoform0023427 | HMGR | 4.76 | 5.12 | 32.67 |
| Isoform0024808 | HMGR | 13.50 | 22.13 | 41.04 |
| Isoform0028006 | HMGR | 11.32 | 20.42 | 30.32 |
| Isoform0028901 | HMGR | 9.07 | 9.61 | 28.72 |
| Isoform0029409 | HMGR | 6.31 | 8.81 | 13.91 |
| Isoform0030063 | HMGR | 1.25 | 2.09 | 42.38 |
| Isoform0030914 | HMGR | 9.41 | 15.27 | 28.05 |
| Isoform0023062 | MVK | 4.36 | 5.45 | 8.06 |
| Isoform0024471 | MVK | 7.54 | 9.04 | 13.71 |
| Isoform0025105 | MVK | 13.63 | 14.03 | 17.17 |
| Isoform0025712 | MVK | 9.03 | 12.34 | 27.41 |
| Isoform0028298 | MVK | 7.98 | 11.37 | 24.03 |
| Isoform0029153 | MVK | 0.54 | 0.74 | 14.23 |
| Isoform0019015 | PMK | 10.49 | 9.01 | 16.99 |
| Isoform0019080 | PMK | 6.99 | 5.13 | 3.71 |
| Isoform0019615 | PMK | 7.86 | 6.62 | 14.52 |
| Isoform0019657 | PMK | 5.48 | 4.31 | 3.49 |
| Isoform0014733 | MVD | 2.20 | 1.47 | 1.13 |
| Isoform0025690 | MVD | 25.05 | 29.48 | 20.32 |
| Isoform0026511 | MVD | 49.63 | 29.51 | 22.71 |
| Isoform0019309 | FPPS | 29.40 | 15.01 | 4.46 |
| Isoform0028074 | FPPS | 111.55 | 59.08 | 28.73 |
| Isoform0005786 | TPS | 3.73 | 1.67 | 3.29 |
| Isoform0006815 | TPS | 80.43 | 25.86 | 0.11 |
| Isoform0007586 | TPS | 8.75 | 6.92 | 9.15 |
| Isoform0007771 | TPS | 0.01 | 0.29 | 14.89 |
| Isoform0010716 | TPS | 89.76 | 28.01 | 0.12 |
| Isoform0014224 | TPS | 0.00 | 0.86 | 43.44 |
| Isoform0016820 | TPS | 0.09 | 1.87 | 99.64 |
| Isoform0018484 | TPS | 0.03 | 1.98 | 98.20 |
| Isoform0018496 | TPS | 55.42 | 45.68 | 2.30 |
| Isoform0018770 | TPS | 0.03 | 0.63 | 24.97 |
| Isoform0019434 | TPS | 7.36 | 3.61 | 1.58 |
| Isoform0020206 | TPS | 30.12 | 27.62 | 1.10 |
| Isoform0020220 | TPS | 0.10 | 2.17 | 98.21 |
| Isoform0020398 | TPS | 7.20 | 5.73 | 0.22 |
| Isoform0021234 | TPS | 74.64 | 33.90 | 0.23 |
| Isoform0021602 | TPS | 113.86 | 35.16 | 0.15 |
| Isoform0022763 | TPS | 62.18 | 18.49 | 0.09 |
| Isoform0023193 | TPS | 0.06 | 1.86 | 80.09 |
| Isoform0024296 | TPS | 129.98 | 40.31 | 0.23 |
| Isoform0029758 | TPS | 116.59 | 35.59 | 0.15 |
| Isoform0031599 | TPS | 0.16 | 1.50 | 83.70 |
| Isoform0035578 | TPS | 13.94 | 10.65 | 11.42 |

**Supplementary Table S11.** DXS proteins from other species used in phylogenetic analysis.

| Protein | Protein ID in NCBI | Species |
| --- | --- | --- |
| AaDXS | ABK35283.1 | *Adonis aestivalis* |
| AmDXS | AID51428.1 | *Astragalus membranaceus* |
| AtDXS3 | AED91669.1 | *Arabidopsis thaliana* |
| CiDXS | XP_042967090.1 | *Carya illinoinensis* |
| CsDXS | ANB66337.1 | *Camellia sinensis* |
| GbDXS1 | AAS89341.1 | *Ginkgo biloba* |
| GbDXS2 | AAR95699.1 | *Ginkgo biloba* |
| HbDXS1 | XP_021635684.1 | *Hevea brasiliensis* |
| HbDXS2 | ABF18929.1 | *Hevea brasiliensis* |
| JrDXS | XP_018826015.1 | *Juglans regia* |
| LaDXS | AGQ04154.1 | *Lavandula angustifolia* |
| MtDXS1 | CAD22530.1 | *Medicago truncatula* |
| MtDXS2 | CAN89181.1 | *Medicago truncatula* |
| PdDXS1 | ACC54557.1 | *Pinus densiflora* |
| PdDXS2 | ACC54554.1 | *Pinus densiflora* |
| PpDXS | XP_007225201.1 | *Prunus persica* |
| QsDXS | XP_023903287.1 | *Quercus suber* |
| RcDXS1 | EEF45457.1 | *Ricinus communis* |
| RcDXS2 | XP_024182614.1 | *Rosa chinensis* |
| RcDXS3 | XP_002514364.1 | *Ricinus communis* |
| SmDXS1 | ACF21004.1 | *Salvia miltiorrhiza* |
| SmDXS2 | ACQ66107.1 | *Salvia miltiorrhiza* |
| TwDXS2 | AKP20998.1 | *Tripterygium wilfordii* |
| VrDXS | XP_034684391.1 | *Vitis riparia* |
| VvDXS2 | XP_002266925.1 | *Vitis vinifera* |
| ZmDXS | ABP88134.1 | *Zea mays* |

**Supplementary Table S12.** GPPS proteins from other species used in phylogenetic analysis.

| Protein | Protein ID in NCBI | Sspecies |
| --- | --- | --- |
| AiGPPS2 | AIG15448.1 | *Azadirachta indica* |
| AmLSU | AAS82860.1 | *Antirrhinum majus* |
| AmSSU | AAS82859.1 | *Antirrhinum majus* |
| AtSPPS2 | AT1G17050 | *Arabidopsis thaliana* |
| CaGPPS | ATZ76916.1 | *Camptotheca acuminata* |
| CfSSU | XP_059662477.1 | *Cornus florida* |
| CrSSU1 | AGL91646.1 | *Catharanthus roseus* |
| CsLSU | ARE72269.1 | *Cannabis sativa* |
| CsGPPS | CAC16851.1 | *Citrus sinensis* |
| HbSSU | XP_021692305.2 | *Hevea brasiliensis* |
| HlLSU | ACQ90682.1 | *Humulus lupulus* |
| LcSSU1 | ART33311.1 | *Litsea cubeba* |
| LcSSU2 | ALT07954.1 | *Leucosceptrum canum* |
| MpLSU | ABW86879.1 | *Mentha piperita* |
| MpSSU | ABW86880.1 | *Mentha piperita* |
| NrSSU2 | QIQ55971.1 | *Nepeta rtanjensis* |
| OsSSU2 | BAF09598.2 | *Oryza sativa* |
| PbSSU2 | APX64489.1 | *Phalaenopsis bellina* |
| PtSSU | QDJ94097.1 | *Populus trichocarpa* |
| QsSSU | XP_023888195.1 | *Quercus suber* |
| SgGPPS | AEM42978.1 | *Siraitia grosvenorii* |
| SmSSU2 | AEZ55680.1 | *Salvia miltiorrhiza* |
| TcGPPS1 | EOY27601.1 | *Theobroma cacao* |
| VvGPPS | AAR08151.1 | *Vitis vinifera* |

**Supplementary Table S13.** TPS proteins from other species used in phylogenetic analysis.

| Protein | Protein ID in NCBI | Species |
| --- | --- | --- |
| AaLINS | ADD81294.1 | *Actinidia arguta* |
| AeNer1 | XP_057488419.1 | *Actinidia eriantha* |
| AgPIN1 | O24475.1 | *Abies grandis* |
| AtTPS02 | AT4G16730 | *Arabidopsis thaliana* |
| AtTPS03 | AT4G16740 | *Arabidopsis thaliana* |
| AtTPS10 | AT2G24210 | *Arabidopsis thaliana* |
| AtTPS14 | AT1G61680 | *Arabidopsis thaliana* |
| AtTPS23 | AT3G25830 | *Arabidopsis thaliana* |
| AtTPS24 | AT3G25810 | *Arabidopsis thaliana* |
| AtTPS27 | AT3G25820 | *Arabidopsis thaliana* |
| AtTPS32 | AT1G79460 | *Arabidopsis thaliana* |
| AtTPS31 | AT4G02780 | *Arabidopsis thaliana* |
| CjLIMS | QBK56509.1 | *Citrus japonica* |
| CsRLIMS1 | XP_015385962.2 | *Citrus sinensis* |
| DcTPS10 | XP_020702845.1 | *Dendrobium catenatum* |
| FhTPS4 | WKC16865.1 | *Freesia hybrida* |
| HcTPS7 | AHJ57305.1 | *Hedychium coronarium* |
| HcTPS8 | AGY49283.1 | *Hedychium coronarium* |
| LaCARS | AGL98419.1 | *Lavandula angustifolia* |
| LaGERDS | AGL98420.1 | *Lavandula angustifolia* |
| LaLINS | Q2XSC5.1 | *Lavandula angustifolia* |
| LfTPS01 | AIO10964.1 | *Liquidambar formosana* |
| MaGERS | KAJ4729952.1 | *Melia azedarach* |
| MosesquiTPS | XP_057947853.1 | *Malania oleifera* |
| MtTPS15 | XP 003621227.1 | *Medicago truncatula* |
| MtTPS23 | XP 003619707.1 | *Medicago truncatula* |
| OsTPS | ABA98308.2 | *Oryza sativa* |
| PgTPS | AFJ73549.1 | *Pinus greggii* |
| QsGERDS | XP_023905164.1 | *Quercus suber* |
| RcGERDS | XP_024161123.1 | *Rosa chinensis* |
| RdGERS | QGW08887.1 | *Rosa damascena* |
| SlTPS5 | NP 001233805.1 | *Solanum lycopersicum* |
| SlTPS8 | XP 004231365.1 | *Solanum lycopersicum* |
| SlTPS40 | NP 001234008.2 | *Solanum lycopersicum* |
| SmTPS | EFJ33479.1 | *Selaginella moellendorffii* |
| VrGERDS | XP_034678034.1 | *Vitis riparia* |
| VrTERS | XP_034705111.1 | *Vitis riparia* |
| VvGwBer | ADR74195.2 | *Vitis vinifera* |
| VvGwGerA | ADR66821.1 | *Vitis vinifera* |
| VvGwgCad | ADR74199.1 | *Vitis vinifera* |
| VvNER1 | RVW86385.1 | *Vitis vinifera* |
| VvOCIS | ADR74206.1 | *Vitis vinifera* |
| VvTERS | AAS79352.1 | *Vitis vinifera* |
| ZmTPS6 | NP 001105674.1 | *Zea mays* |
| ZmTPS10 | NP 001105850.1 | *Zea mays* |
| ZmTPS23 | ABY79213.1 | *Zea mays* |
